# Tripartite motif containing 69 elicits ERK2-dependent EYA4 turnover to impart pancreatic tumorigenesis

**DOI:** 10.7150/jca.79905

**Published:** 2023-01-01

**Authors:** Yu Jia, Hui-Yan Li, Jue Wang, Xing Chen, Lu Lou, Yan-Yan Wei, Ying Wang, Shi-Jing Mo

**Affiliations:** 1 Cancer Research Center, Tongji Hospital, Tongji Medical College, Huazhong University of Science and Technology, Wuhan 430030, Hubei, P.R. China.; 2 General Surgical Laboratory, The First Affiliated Hospital, Sun Yat-Sen University, Guangzhou, 510080, Guangdong, P.R. China.; 3 Department of Pathology, The First Affiliated Hospital, Sun Yet-Sen University, Guangzhou 510080, Guangdong, P.R. China.; 4 Department of Surgery, Huashan Hospital, Fudan University, Shanghai 200040, P.R. China.

**Keywords:** Tripartite motif containing 69, Eyes absent homologue 4, Turnover, Pancreatic ductal adenocarcinoma

## Abstract

Eyes absent homologue 4 (EYA4) is silenced in pancreatic ductal adenocarcinoma (PDAC) and functions as a tumor suppressor to restrain PDAC development, albeit the molecular mechanism underlying its downregulation remains enigmatic.

**Methods:** Functional studies were determined by immunohistochemistry of PDAC samples from patients and *Pdx1*-Cre; LSL-Kras^G12D/+^; Trp53^fl/+^ (KPC) mice, three-dimensional spheroid culture, flow cytometry, MTT and subcutaneous xenograft experiments. Mechanistical studies were examined by cellular ubiquitination, cycloheximide (CHX) pulse-chase, co-immunoprecipitation, chromatin immunoprecipitation, GST-pulldown, *in vitro* protein kinase assay, immunofluorescence and luciferase reporter assays.

**Results:** We screen E3 ligase that is negatively correlated with EYA4 and uncover a mutually exclusive interaction of tripartite motif containing 69 (TRIM69) with EYA4 in human PDAC. TRIM69 elicits EYA4 polyubiquitylation and turnover independent of P53 and impedes the EYA4-driven deactivation of β-catenin/ID2 cascade, fueling PDAC cell proliferation *in vitro* and tumor development in mice. Expression of TRIM69 is upregulated in PDAC samples from independent cohorts of patients and the *Pdx1*-Cre; LSL-Kras^G12D/+^; Trp53^fl/+^ (KPC) mice, and associated with unfavorable prognosis. Depleting TRIM69 preferentially induces lethality in the EYA4-deficient PDAC cells. We further unearth that ERK2 directly binds to the D-site of mitogen-activated protein kinase (MAPK) docking groove in EYA4 Leu512/514 and phosphorylates EYA4 at Ser37, which is instrumental for EYA4 polyubiquitylation and turnover by TRIM69.

**Conclusion:** Our results define a previously unappreciated role of TRIM69-EYA4 axis in pancreatic tumorigenesis and underscore that targeting TRIM69 might be an effective therapeutic approach for PDAC harboring EYA4 deficiency.

## Introduction

Eyes absent homologue 4 (EYA4), encoded by *eya4* gene, whose primary function is responsible for eye development in *Drosophila* and postlingual sensorineural hearing loss, has been proposed as a tumour suppressor with frequent inactivating mutations and deletions in a variety of sporadic tumors 1, 2. Although EYA4 may in some cases boost tumorigenic phenotype such as epithelial-mesenchymal transition (EMT) 3, accumulating evidence suggest that EYA4 has crucial tumor-suppressive role in both human and experimental mice malignancies. For instance, overexpression of EYA4 suppresses the c-JUN-mediated angiogenesis and metastasis of hepatocellular carcinoma (HCC) in nude mice 4. It is documented that EYA4 inhibits growth and metastasis of bladder cancer cells mediated by circular RNA (circRNA) ACVR2A-miR-626 axis 5. The multifaceted role of EYA4 has been linked to the transcriptional mechanism whereby EYA4 coordinately interacts with sine oculis homeobox (SIX) and dachshund family transcription factor 1 (DACH) through its C-terminal domain (ED) and the posttranslational modification by which EYA4 evokes intracellular signaling transduction via its N-terminal domain (NTD) 6. Our recent study demonstrated that EYA4 is silenced in pancreatic ductal adenocarcinoma (PDAC) and forced expression of EYA4 can block nuclear β-catenin inclusion and *Id2* transcription to oppose PDAC development 7. Additionally, EYA4 deficiency correlates with tumour malignancy and poor prognosis in HCC 8. Despite extensive researches on identification of EYA4 as a tumor suppressor, the mechanism underlying its deficiency in PDAC remains poorly understood.

Ubiquitination, like other types of posttranslational modifications (e.g., phosphorylation, acetylation, nitrosylation, oxidation and glycosylation), plays a principal role in numerous pathophysiological processes including organogenesis, innate/adaptive immune response, cell apoptosis and tumor development 9. The Lys^48^-linked polyubiquitylation provides a signal for the proteasome-mediated degradation of substrate protein, yet the Lys^63^-linked polyubiquitylation is predominantly responsible for spatial protein trafficking. Tripartite motif containing 69 (TRIM69) belongs to the evolutionarily conserved TRIM E3 ligase family that catalyzes substrate polyubiquitylation via its N-terminal RING finger domain 10. The biological function of TRIM69 is just beginning to be uncovered to date. TRIM69 has been implicated in brain development of zebrafish and found to be an interferon stimulated gene (ISG) with anti-virus activity 11, 12. TRIM69 defect exaggerates inflammatory damage and neuron death 13. TRIM69 inhibits apoptosis of human lens epithelial cells (HLECs) by sustaining P53 polyubiquitylation and turnover 14, 15. Despite advancement in understanding the dynamic balance in molecular interaction of TRIM69, the functional significance of TRIM69 in PDAC development is largely unknown.

Aberrant activation of extracellular regulated protein kinase (ERK) represents the remarkable oncogenic event in PDAC 16, 17; however, the linkage between ERK and EYA4, particularly whether and how ERK orchestrates EYA4 in pathogenesis of PDAC remains undefined. Here we conduct a cancer cell line encyclopedia (CCLE)-based screen across 40 genes encoding E3 ligase in 12 pancreatic carcinoma cell lines to identify the candidate whose expression is inversely correlated with EYA4. In this analysis, we identify TRIM69 as a mutually exclusive E3 ligase that elicits EYA4 polyubiquitylation and turnover independent of P53. Mechanistically, ERK2 directly binds to the D-site of mitogen-activated protein kinase (MAPK) docking groove in EYA4 Leu512/514 and phosphorylates EYA4 at Ser37, which is required for EYA4 polyubiquitylation and turnover by TRIM69. We characterize the inhibitory role of TRIM69 in the EYA4-driven tumor suppression through genetic and biochemical approaches as well as *in vitro* and *in vivo* studies. We also provide evidence that high expression of TRIM69 in PDAC is an independent predictor of tumor aggressiveness with significant hazard ratios for predicting clinical outcome. Targeting TRIM69 preferentially kills the EYA4-deficient PDAC cells, which can be further investigated clinically with small-molecule inhibitor.

## Materials and Methods

### Human subjects

One-hundred-fifteen patient samples used in this study were obtained from patients undergoing surgery for PDAC at the First Affiliated Hospital of Sun Yat-Sen University (SYSU) and examined by a general pathologist to confirm histological diagnosis and verify the high-density cancer foci in the selected samples. The inclusion criteria were: (1) availability of tumor specimens, (2) curative resection and (3) integrated clinicopathological and follow-up data. The exclusion criteria included: (1) receiving preoperative neoadjuvant therapy and (2) died within 30 days after pancreatectomy. The clinicopathological features of these PDAC patients were presented in Supplemental Table 1. Immunohistochemistry (IHC) analyses were performed on 115 paraffin-embedded PDAC tissues to evaluate the correlation of TRIM69 with EYA4 expression and clinicopathological features of PDAC patients. Overall survival (OS) was the interval between dates of surgery and death. Recurrence-free survival (RFS) was the interval between dates of surgery and recurrence (if recurrence was not diagnosed, patients were classified on the date of death or the last follow-up). The total of 10 PDAC tissues of patients for TRIM69 and EYA4 quantification by western-blotting were obtained immediately after surgical resection, snap frozen in liquid nitrogen and stored at -80 °C until protein extraction. The 30 paired PDAC specimens and their adjacent non-tumorigenic specimens in which RNAs are available were as previously described 7. All samples were collected following informed consent in accordance with the policy of internal review and ethics boards from the First Affiliated Hospital of SYSU.

### Mice

All animal experiments were conducted with the approval of the Institutional Animal Care and Use Committee (IACUC) guidance of Huazhong University of Science and Technology (HUST). The KPC (*Pdx1*-Cre; LSL-Kras^G12D/+^; Trp53^fl/+^) mice which could spontaneously develop pancreas adenocarcinoma were generated by crossing the *Pdx1*-Cre mice with the LSL-Kras^G12D/+^;Trp53^fl/+^ (KP) mice as in previous publication^18^. All mice were bred in a specific-pathogen-free environment, with the temperature maintained at 24 °C and sterile conditions at the Animal Experiment Center of HUST.

### Immunohistochemistry

Immunohistochemical stainings were performed on 5-µm tissue sections as reported in previous studies 7, 18, 19. In brief, the slides were deparaffinized by baking slides at 60 °C for 30 min and then rehydrated in series of ethanol solutions. The endogenous peroxidase activities were quenched by 3% H_2_O_2_ in distilled water for 30 min. Following three washes with TT buffer (500 mmol/L NaCl, 10 mmol/L Trizma, and 0.05% Tween-20), antigen retrieval was processed by microwave heating for 20 min in 10 mmol/L citrate-based antigen unmasking solution. Slides were blocked with 5% BSA in TT buffer for 1 h after cooling down to room temperature and incubated with the primary antibodies at 4 °C overnight. After three washes with TT buffer, secondary antibody was added into slides for 1 h, followed by detection of Dako ChemMate^TM^ Envision^TM^ Detetcion Kit (DaKo, Glostrup, Denmark). The images were captured using Nikon Ti-S microscope equipped with a digital camera system (Nikon, Japan) and evaluated independently by two experienced observers who were blinded to the data.

### Reagents, plasmids and antibodies

MG132 (#S2619), Bortezomib (#S1013), SCH772984 (#S7101) and RITA (#S2781) were purchased from Selleck Chemicals LLC (Houston, TX). Cycloheximide (#HY-12320) was purchased from MedChem Express (MCE, Shanghai, China). Recombinant human EGF (#SRP3027) and Wnt3a (#H17001) were ordered from Sigma-Aldrich (St. Louis, MO). Ni-NTA agarose (#R901-15) was from Invitrogen (Waltham, MA). The GFP-tagged wild-type EYA4 plasmid and shRNA duplexes targeting EYA4 were constructed as previously described 7, 8. Full length TRIM69^WT^ (1-500) and truncated TRIM69^ΔR^ (1-41, 82-500) were acquired as described elsewhere 10 and subcloned into c-Flag pcDNA3 vector (#20011, Addgene). The EYA4 L512/514A, S37A, and S37E mutants were constructed using a QuickChange^®^ Site-Directed Mutagenesis Kit (Agilent Technologies, Santa Clara, CA). The GST-tagged ERK2 was subcloned into the pGEX-4T-2 vector. The luciferase reporter plasmids containing wild-type (CCTTTGATC; TOP-FLASH) or mutant (CCTTTGGCC; FOP-FLASH) TCF/LEF DNA binding sequence were purchased from Upstate Biotechnology (Lake Placid, NY). The pLKO.1-puro lentiviral shRNA oligonucleotides targeting TRIM69 were: CCGGTGGCAACCAGAGAGCTTATTTCTCGAGAAATAAGCTCTCTGGTTGC CATTTTTG. The siRNAs targeting TRIM13, 37, 25 and 69 were synthesized by GenePharma (Shanghai, China), respectively. P53 shRNA (#sc-29435-V), PKA siRNA (#sc-36240) and non-specific control siRNA (#sc-37007) were purchased from Santa Cruz Biotechnology (Santa Cruz, CA). Antibodies used in this study were: EYA4 (#ab93865, Abcam), TRIM69 (#ab111943, Abcam), TRIM13 (#ab234847, Abcam), TRIM37 (#ab264190, Abcam), TRIM25 (#ab86365, Abcam), P53 (#2524S, Cell Signaling Technology), PUMA (#24633S, Cell Signaling Technology), P27 (#3686, Cell Signaling Technology), phospho-ERK1/2 T202/Y204 (#4370S, Cell Signaling Technology), ERK1/2 (#4695S, Cell Signaling Technology), GFP (#MAB2510, Sigma-Aldrich), Ub (#3933S, Cell Signaling Technology), phospho-β-catenin S675 (#4176S, Cell Signaling Technology), β-catenin (#8480S, Cell Signaling Technology), p-Ser (#ab9332, Abcam), Flag (#F7425, Sigma-Aldrich), α-PKA (#4782S, Cell Signaling Technology), ID2 (sc-398104, Santa Cruz Biotechnology), RAS (#3965S, Cell Signaling Technology), c-RAF (#53745S, Cell Signaling Technology), MEK1/2 (#8727S, Cell Signaling Technology), Ki-67 (#NB500-170, Novus Biologicals), Lamin B (#bs-1840R, Biosynthesis), GAPDH (#bs-0755R, Biosynthesis) and β-actin (#bsm-33036M, Biosynthesis).

### Cell culture and transfection

SW-1990 cells, Capan-2 cells and the pancreatic patient-derived cancer cells (PDC#0183) were cultured as described previously 18. T3M-4 PDAC cells were cultured in Dulbecco's modified Eagle's medium (DMEM) supplementing with 10% fetal bovine serum (FBS), glutaMAXTM-I and penicillin (100 U/mL) /streptomycin (0.1 mg/mL). Human pancreatic ductal epithelial (HPDE) cells were grown in RPMI-1640 medium (Gibco, Waltham, MA, USA) with 10% FBS at 37 °C with 5% CO_2_. All cell lines were routinely tested for mycoplasma and found to be negative. Transient transfection were performed using Lipofectamine 3000 reagent (Invitrogen, Carlsbad, CA, USA) as previously described 18, 19. Cells were harvested at 48 hours after transfection and the efficacy of RNA interference was evaluated by western-blotting and RT-qPCR, respectively. To package lentivirus, pLKO.1-puro plasmid was cotransfected into human embryonic kidney 293T (HEK293T) cells with four helper plasmids (pHDM-VSV-G, pHDM-tat1b, pHDM-HgPM2, and pRC-CMVRaII). The viral supernatant was harvested at 72 h after transfection followed by passing through a 0.45 μm filter. PDAC cells up to 60% confluence were incubated in lentivirus-containing medium with 10 μg/mL polybrene (Sigma-Aldrich). Forty-eight hours later, cells were selected by 1 μg/mL puromycin (Invitrogen).

### TRIM69 knockout by CRISPR-Cas9

For *Trim69* gene knockout (KO), the commercial lenti-pCRISPR-LvSG03 vector carrying human TRIM69 gRNA (LPPHCP271544L03-1-100) from Genecopoeia (Rockville, MD) was transfected into SW-1990, Capan-2 and T3M-4 cells, respectively. After selecting with 1 μg/mL puromycin for two days, cells were transferred into fresh medium and seeded at low density to allow single colony growth. Colonies were then picked for KO validation by western-blotting or real-time quantitative PCR (RT-qPCR).

### Three-dimensional spheroid culture

For 3D spheroids culture assay, the indicated PDAC cells were cultured in 48-well plates at a density of 200 cells per well for 48 h at 37 °C before embedding in BD BioCoat Matrigel^TM^ (#356230). After completed gelation, 250 μL of culture medium was added and the culture medium was changed every three days. The numbers of the spheroids were counted at 7-10 days.

### Flow cytometry with active Caspase-3 staining

For detection of active Caspase3 (aCASP3)-positive cells by flow cytometry, 20, 000 of the indicated PDAC cells were fixed with 1:4 Fixation/Permeabilization Concentrate in Fixation/Permeabilization Diluent (#00-5521-00, eBioscience^TM^) overnight at 4 °C. Next day cells were washed with permeabilization buffer (#00-8333-56, eBioscience^TM^) and incubated for 1 h at 4 °C with 5 μL of the Alexa Fluor^®^ 647-conjugated antibodies against aCASP3 (#560626, BD Biosciences). After washing with PBS, signals were determined by flow cytometry.

### Glutathione S-transferase pull-down assay

Glutathione S-transferase (GST) pull-dwon assay was conducted as described in previous publications using Pierce^TM^ GST Protein Interaction Pull-Down Kit (#21516, Thermo) according to the manufacturer's protocol 18, 19. In brief, the 50 μg indicated “prey” proteins were precleared with 50 μL prewashed glutathione agarose in 500 μL GST pull-down buffer (20 mmol/L Tris, 150 mmol/L NaCl, 2 mmol/L MgCl_2_, 0.1% NP-40 and 20 μg/mL BSA) for 1 h at 4 °C. The 100 μg indicated GST-tagged “bait” proteins were incubated with 50 μL prewashed glutathione agarose in 500 μL GST pull-down buffer for 1 h at 4 °C. The 'bait-agarose' solutions were combined with precleared 'prey' solutions and mixed for additional 2 h at 4 °C. Protein-bound agarose was washed 3 times and boiled in sodium dodecylsulfate (SDS) sample buffer. Supernatants were then subjected to SDS-polyacrylimide gel electrophoresis (PAGE) and western-blotting analysis.

### *In vitro* protein kinase assay

The *in vitro* protein kinase assay was performed as described previously with some modifications 19. In brief, vectors expressing GFP-tagged wild-type EYA4 or mutant EYA4 S37A were transfected into SW-1990 cells, respectively and the cell lysates were incubated the GFP-tagged beads overnight. The purified WT EYA4 and mutant EYA4 S37A were then incubated with recombinant ERK2 protein (#M28-10G, SignalChem) in the presence of 50 mM adenosine triphosphate for 30 min. The reaction products were resolved in SDS-PAGE and detected by western-blotting.

### Cellular ubiquitination assay

*In vivo* ubiquitination assay was performed as previously described 18.

Briefly, the indicated PDAC cells were transfected with His-tagged ubiquitin and lysed by radioimmunoprecipitation assay (RIPA) buffer (50 mmol/L Tris HCl at pH 7.4, 150 mmol/L NaCl, 1 mmol/L EDTA, 1% Nonidet P-40 and 10% glycerol) containing protease and phosphatase inhibitors, followed by nickel bead purification and western-blotting analyses. For *in vitro* ubiquitination assay, Flag-TRIM69 were transfected into HEK293T cells and eluted by Flag peptides according to manufacturers' standard procedures. Purified GFP-EYA4 and Flag-TRIM69 proteins were incubated at 37 °C for 2 h in the presence of 20 μL reaction buffer (20 mmol/L Tris-HCl, 5 mmol/L MgCl_2_, 50 mmol/L NaCl, 1 mmol/L 2-mercaptoethanol and 10% glycerol) supplementing with 50 µmol/L ubiquitin, 50 nmol/L E1, 2 µmol/L E2 (UbcH5c), 2 µmol/L CHIP, 2 µmol/L Hsp70 and 2 mmol/L ATP (#J5110, UBPBio). After terminating the reactions with RIPA buffer, the samples were fractionated by 10% SDS-PAGE for western-blotting analysis.

### Cycloheximide (CHX) pulse-chase assay

CHX pulse-chase assay was performed when PDAC cells reached about 60% confluence in 2-cm plates. Plasmids bearing wild-type TRIM69 or sgRNA were utilized for transfection as indicated in individual experiments. Forty-eight hours later, cells were treated with the protein synthesis inhibitor 20 μg/mL CHX for the indicated duration before collection. The total protein lysates were resolved in SDS-PAGE and subjected to western-blotting.

### Subcellular fractionation

SW-1990 cells stably expressing GFP-EYA4 were transfected with the indicated plasmids and stimulated with Wnt3a (20 ng/mL) for 12 h before being collected. Cytosolic and nuclear fractionations were eluted using NE-PER^TM^ Nuclear and Cytoplasmic Extraction Reagents Kits (#78835, Thermo) according to the manufacturer's guideline.

### Immunofluorescence

The procedure for immunofluorescence (IF) was described in previous publications 18, 19. Briefly, cells grown on coverslips were fixed with 4% paraformaldehyde (PFA), permeated with 0.3% Triton X-100 and blocked with 1% BSA, followed by incubation with a mixture of anti-β-catenin primary antibody at 4 °C overnight, and a mixture of secondary antibody (Alexa Fluor^®^ 594, Cell Signaling Technology) in the dark for 1 h. For xenograft IF staining, the paraffin-embedded tumor tissues were sectioned to 4 μm thickness, deparaffinized with dimethylbenzene, antigen-retrieved by EDTA antigen retrieval buffer (pH 8.0), blocked in 5% BSA for 30 min, permeabilized in 0.2% Triton X-100 for 15 min and incubated with the primary antibody targeting Ki-67 (#NB500-170, Novus Biologicals). Nuclei were counterstained by 4′,6-diamidino-2-phenylindole (DAPI) (#KGR0001, KeyGene Biotech) and pictures were captured using an Axio Imager2 microscope (Carl Zeiss, Germany).

### Co-immunoprecipitation

Co-immunoprecipitation was carried out using the protocol reported previously 18, 19. In brief, the indicated PDAC cells were lysed in RIPA buffer for 1 h, followed by incubating with protein A/G-agarose beads (Cwbiotech, Beijing, China) and the primary antibody at 4 °C overnight. The beads were subsequently washed three times with washing buffer (50 mmol/L Tris-HCl at pH 7.6, 300 mmol/L NaCl, 1 mmol/L EDTA, 0.5% NP-40 and 10% glycerol), boiled for 10 min in SDS-sample buffer and analyzed by western-blotting.

### Western-blotting

Sample preparation of cell lysates, immuneprecipitants, SDS-PAGE, membrane transfer and blotting were as previously described 18, 19. In brief, cell lysates or immuneprecipitants were subjected to SDS-PAGE and proteins were transferred to Immobilon^TM^ PVDF Transfer Membranes (Millipore Corporation, Billerica, MA). The membranes were blocked in Tris-buffered saline (TBS) containing 5% bovine serum albumin (BSA), washed twice in TBS containing 0.1% Tween-20, and incubated with primary antibody overnight at 4 °C, followed by secondary antibody for 1 h at room temperature. The proteins of interest were visualized using western chemiluminscent HRP substrate kit (PPLYGEN, Beijing, China).

### Chromatin immunoprecipitation (ChIP) assay

ChIP assays were performed using EZ-Magna ChIP kit (#17-408, Millipore, Billerica, MA) as described previously 18, 19. In brief, three million SW-1990 cells stably expressing GFP-EYA4 were transfected with Flag-TRIM69, followed by 20 ng/mL Wnt-3a stimuli for 12 h and 1% formaldehyde cross-link for 15 min at 37 °C. The cross-linking reaction was quenched by glycine and cells were lysed in SDS buffer containing protease inhibitor cocktail. Cell lysates were sonicated to shear chromatin DNA into fragments with 200-1000 base pairs in size and immunoprecipitated with 5 μg anti-IgG or anti-β-catenin antibodies. After decrosslinking of immunoprecipitated DNA fragments at 65 °C in high-salt condition, the purified DNAs were analysed by semi-PCR with the following primers:

- *Id2*: 5′-TCTTAAAGCCAGGACCCCAG-3′ (forward) and,
- 5′-TTCGCGCCCTCATTACTACT-3′ (reverse).

### Luciferase reporter assay

The luciferase reporter assay was conducted using a Dual-Luciferase^®^ Reporter (DLR^TM^) Assay System (#E1910, Promega) according to the manufacturer's instruction. Cells were seeded in triplicate in 96-well plates and allowed to settle for 24 hours. The indicated luciferase reporter plasmids plus 1 ng pRL-TK *Renilla* plasmid were transfected into the cells using Lipofectamine 3000 reagent. Cells were harvested and their luciferase activities were measured forty-eight hours later as previously reported 18, 19.

### Real-time quantitative PCR (RT-qPCR)

Total RNA was extracted with TRIzol (#15596018, Invitrogen) and reverse-transcribed to complementary DNA by PrimeScript^®^ RT reagent Kit (Takana, Dalian) using Super Array PCR master mix (SuperArray Bioscience, Frederick, Maryland) as described previously 7, 8, 18, 19. Real-time PCR was run with the double-stranded DNA dye SYBR Green PCR Mastermix in Takana SYBR^®^ Premix Ex Taq^TM^ Kit (Takana, Dalian) following the manufacturer's instruction. Real-time monitoring of PCR amplification was performed using the LightCycler^®^ 480 detection system (Roche). Data were expressed as relative mRNA levels normalized to GAPDH expression level in each sample and represented as mean ± s.d. of at least three independent experiments. The primer sequences are listed as below:

- *Trim69* sense: 5′-CTTGCCATCCAACAGGGTCAA-3′;
- *Trim69* antisense: 5′-TTCCTTGTGAGCAGCAATAGC-3′;
- *Trim13* sense: 5'-GTTTTGCCTTGCTCCCACAAC-3';
- *Trim13* antisense: 5'-TCCTTACGGCATGTAGGACAC-3';
- *Trim37* sense: 5'-TATGGAGAAATTGCGGGATGC-3';
- *Trim37* antisense: 5'-GTCAGCCAGCGCCTAATACAG-3';
- *Trim25* sense: 5'-AATCGGCTGCGGGAATTTTTC-3';
- *Trim25* antisense: 5'-TCTCACATCATCCAGTGCTCT-3';
- *Eya4* sense: 5'-GAATAACACAGCCGATGG-3';
- *Eya4* antisense: 5'-CCAGGTCACTATCAGGAG-3';
- *Id2* sense: 5'-AGTCCCGTGAGGTCCGTTAG-3';
- *Id2* antisense: 5'-AGTCGTTCATGTTGTATAGCAGG-3';
- *P53* sense: 5'-CAGCACATGACGGAGGTTGT-3';
- *P53* antisense: 5'-TCATCCAAATACTCCACACGC-3';
- *Bbc3* sense: 5'-GACCTCAACGCACAGTACGAG-3';
- *Bbc3* antisense: 5'-AGGAGTCCCATGATGAGATTGT-3';
- *Cdkn1b* sense: 5'-AACGTGCGAGTGTCTAACGG-3';
- *Cdkn1b* antisense: 5'-CCCTCTAGGGGTTTGTGATTCT-3';
- *Gapdh* sense: 5'-AATCCCATCACCATCTTCCA-3';
- *Gapdh* antisense: 5'-TGGACTCCACGACGTACTCA-3'.

### Trypan blue assay

Trypan blue staining was performed in PDAC cells expressing GFP-EYA4 or those coexpressing GFP-EYA4 plus Flag-TRIM69. Briefly, cells were plated in 6-well plates at a density of 5×10^4^ cells per well. At the indicated time points, cells were trypsinized and suspended in 1 × PBS. 100 μL cells and 100 μL trypan blue solution (#302643, Sigma-Aldrich) were mixed and the number of viable cells was measured using the Bio-Rad automated cell counter.

### Cell viability assay

Cell viability of the TRIM69-KO PDAC cells was measured by 3-(4, 5-dimethylthiazol-2-yl)-2, 5-diphenyltetrazolium bromide (MTT) assay in the presence or absence of EYA4 shRNA transfection for 72 h as described previously 18, 19. MTT solution in 1× PBS was added to each well at the final concentration of 0.5 mg/mL and the plate was incubated for 4 h at 37 °C. The MTT medium was aspirated carefully and the dark-blue formazan was solubilized with dimethylsulfoxide (DMSO). Optical density was measured with a spectrometer at 490 nm by spectrometer (Wellscan MK3; Labsystems Dragon). Each experiment was made in triplicates and repeated independently for at least three times.

### Animal studies

Mice were maintained in a specific pathogen-free facility and xenograft models were established as described previously 7, 8, 18, 19. In brief, the indicated PDAC cells (1 × 10^7^) suspended in 200 µL PBS were inoculated subcutaneously into right flank of BALB/c athymic nude mice (*nu/nu*, male). The recipient mice were randomized to groups comprised of approximately equal numbers of mice after inoculation. Tumor volumes were measured at the indicated time points and calculated as length×width^2^×0.5. After 35-38 days, mice were sacrificed by cervical dislocation and xenografts were carefully removed, fixed, photographed and weighed. All groups contained at least six mice to support the statistical analysis performed and body weights of mice were measured per five days.

### Molecular docking of ERK2-EYA4 complex

The molecular docking of ERK2-EYA4 complex structure was conducted by HADDOCK algorithm (version 2.2) using ambiguous interaction restraints (AIRs). The primary structures for docking were an X-ray crystal structure of human ERK2 complexed with a MAPK docking peptide (PDB ID: 4H3Q) with resolution 2.2 Å and the modeling structure of EYA4 with residue 369 to 639 constructed from MODELLER(V9.14) 20. E109, T110, L157, N158, T159, Y316, D318 and D321 were included as the active residues of ERK2, while K507, L512 and L514 were included as the active residues of EYA4. All the neighbors of these active residues were selected as passive residues. The structure was selected as a final model of the ERK2-EYA4 complex based on the HADDOCK score.

### Bioinformatics analysis

**Figure 1:**
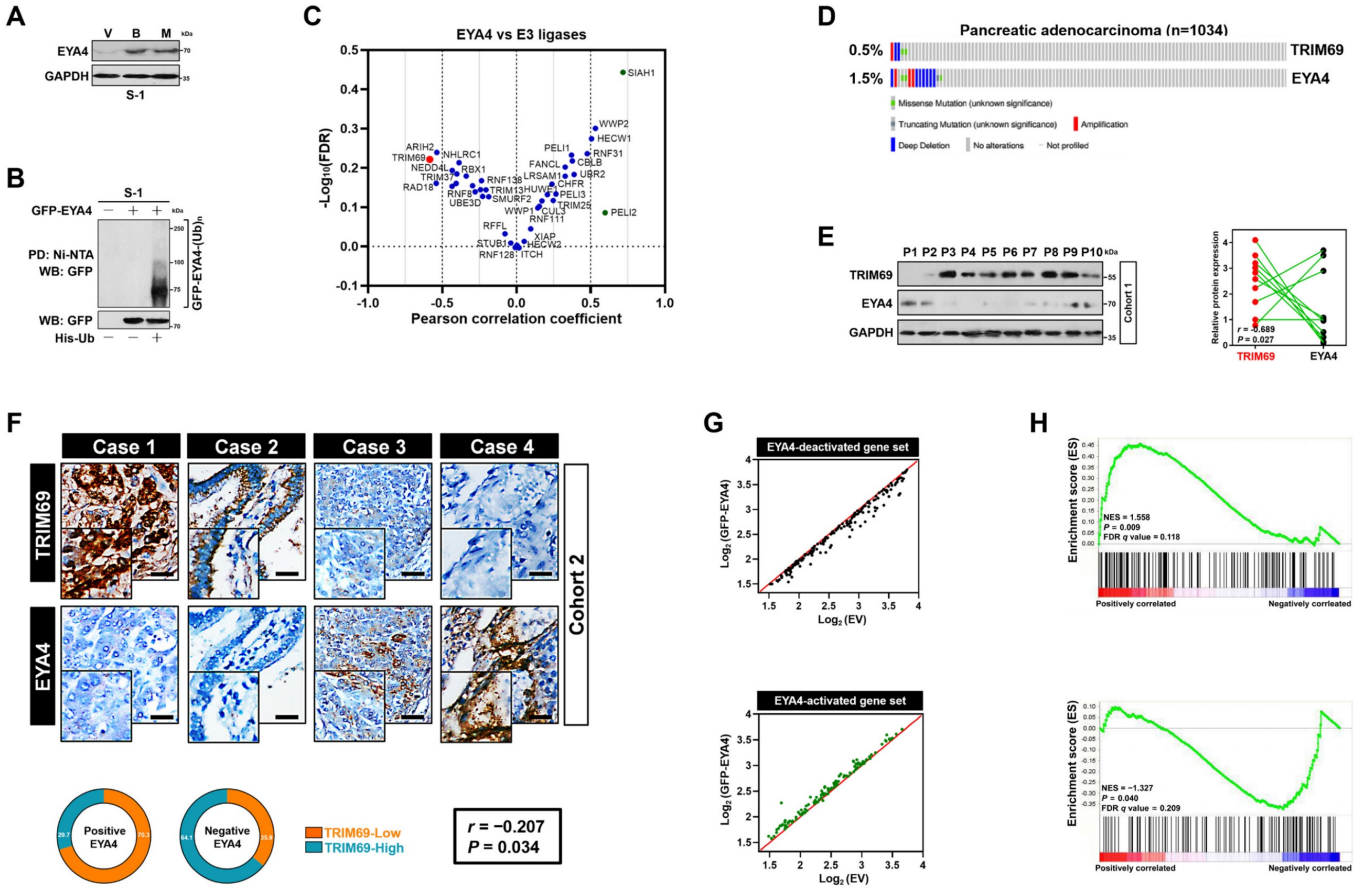
** Identification of the mutually exclusive interaction between TRIM69 and EYA4 in human PDAC. (A)** Western-blotting analyses examining abundance of endogenous EYA4 protein in SW-1990 (S-1) cells treated with Bortezomib (B, 200 nmol/L) or MG132 (M, 20 μmol/L) for 8 h. GAPDH was used as the loading control. WB, western-blotting. **(B)** Cellular ubiquitination assays comparing polyubiquitylation levels of EYA4 in SW-1990 (S-1) cells expressing GFP-tagged wild-type EYA4. PD, pull-down; Ni-NTA, Ni^2+^-nitrilotriacetic acid. **(C)** Correlation analysis for expression of EYA4 versus 40 E3 ligase-encoding genes in pancreatic cancer cell lines from CCLE dataset. **(D)** Genomic alterations of TRIM69 and EYA4 in TCGA PDAC database. **(E)** Western-blotting analyses and Spearman order correlations showing the relationship between TRIM69 and EYA4 in 10 freshly collected, snap-frozen specimens from PDAC patients who had experienced radical resection. **(F)** Representative cases of immunohistochemistry (*top panel*) and pie charts (*bottom panel*) showing the correlation between TRIM69 and EYA4 in 115 primary human PDAC specimens. Scale bar = 50 μm. The *P* value shown was calculated by Spearman order correlations. **(G)** Deactivation (*top panel, n* = 158) or activation (*bottom panel, n* = 116) of gene sets in S-1 cells after ectopic EYA4 expression. Each point represents mRNA levels for a single gene before (x axis) or after (y axis) overexpressing GFP-EYA4. **(H)** Gene set enrichment analysis (GSEA) plot showing the correlation of either EYA4-deactivation (*top panel*) and EYA4-activation (*bottom panel*) gene signature with TRIM69 in GSE55643, respectively.

The correlation between TRIM69 and EYA4 was analyzed in 12 pancreatic cancer cell lines (PATU8988S, SW1990, PANC1, HPAC, SU8686, PANC0203, SNU324 SNU213, PATU8902, DANG, HUPT3 and KP3) from CCLE (https://portals.broadinstitute.org/ccle). Gene expression data from PDAC datasets used for Gene Set Enrichment Analysis (GSEA) in Figure 1H are accessible from GSE55643 deposited in Gene Expression Omnibus (GEO) (http://www.ncbi.nlm.nih.gov/gds/) 21. GSEA was performed using version 4.0 of the molecular signature database (MolSigDB) (http://www.broadinstitute.org/gsea/msigdb/index.jsp). The mRNA expression of TRIM69 in TCGA pan-cancer samples was downloaded from http://www.cbioportal.org. Three datasets (Badea, Ishikawa and Pei) that compared TRIM69 expression between pancreatic cancer and normal pancreatic tissues were identified by searching the Oncomine cancer profiling database 22. Heat maps in Figure 3C were generated from microarray dataset deposited in GEO accession number GSE15471 and GSE16515. The association of “TRIM69 signature” with overall survival or recurrence-free survival of PAAD patients was conducted by Kaplan-Meier Plotter using TCGA database (http://www.kmplot.com/analysis/index.php?p=background).

### Statistics

Statistics were carried out using log-rank test, Bonferroni post hoc t test or student's t-test. Statistical analysis was performed using SPSS for Windows (20.0; SPSS, Inc.) and GraphPad Prism (Prism 8.0; GraphPad Software Inc.) packages. A *P* value < 0.05 was considered significant. Error bars indicate the standard deviation in all the Figures.

## Results

### Identification of the mutually exclusive interaction between TRIM69 and EYA4 in human PDAC

The molecular mechanism underlying EYA4 deficiency in human PDAC remains hitherto unclear. Given the aberrant accumulation of proteasome in cancer cells, we were interested in whether ubiquitin-proteasome system (UPS) can manipulate EYA4 protein turnover. For this purpose, two PDAC cell lines, SW-1990 and Capan-2 (S-1 and C-2 for brevity), which have negligible levels of endogenous EYA4, were treated with bortezomib and MG132, the 20S and 26S proteasome inhibitor, respectively. Either bortezomib or MG132 treatment robustly increased the steady-levels of EYA4 protein―the phenomenon that could also be observed in pancreatic patient-derived cancer cells (PDC), EYA4-proficient T3M-4 (T-4) PDAC cells and the non-tumorigenic human pancreatic ductal epithelial (HPDE) cells (Figure 1A and Supplemental Figure 1A-D), indicating that the posttranslational degradation of EYA4 protein by UPS is a common feature not restricted to PDAC cells. To directly confirm whether EYA4 undergoes ubiquitylation under basal conditions, we examined EYA4 protein status in the stable S-1 and C-2 transfectants bearing GFP-tagged wild-type EYA4 that had been well-established in our previous study 7. Cellular ubiquitination assay of the His-tagged Ub protein immobilized on Ni^2+^-nitrilotriacetic acid (Ni-NTA)-sepharose beads with an anti-GFP antibody showed that EYA4 was poly-ubiquitylated in the two PDAC cell lines (Figure 1B and Supplemental Figure 1A), further validating EYA4 could be degraded through UPS.

To identify potential E3 ligase (s) responsible for EYA4 turnover within PDAC cells in an unbiased manner, we conducted a cancer cell line encyclopedia (CCLE)-based screening approach to compare RNA sequencing data of 40 E3 ubiquitin ligases in a panel of 12 pancreatic carcinoma cell lines for identifying the candidate whose expression is inversely correlated with EYA4. Using Pearson's rank correlation coefficient as the cutoff, we found that some E3 ligases, including CUL3, RNF128, WWP1, MDM2, ITCH, NEDD4L and XIAP had no significant correlation with EYA4, whereas SIAH1, a negative regulator of homeodomain-interacting protein kinase 2 (HIPK2) 23, was positively correlated with EYA4 (*r* = 0.718, *P* = 0.009). PELI2, an isoform of pellino homolog E3 ligase that has a central role in initiating formation of NACHT, LRR and PYD domains-containing protein 3 (NLRP3) inflammasome indispensable for adaptive immune suppression in pancreatic cancer 24, 25, was found to be positively correlated with EYA4 but to a lesser extent than SIAH1 (*r* = 0.597, *P* = 0.041). Conversely, RAD18, which plays a principal role in DNA damage repairment 26; and ARIH2, whose transcription predicts latency of acute myeloid leukemia (AML) 27, tended to be negatively correlated with EYA4 (*r* = -0.541, *P* = 0.069 and *r* = -0.537, *P* = 0.072), yet such correlation did not reach statistical significance. As a validation to our CCLE-based screening strategy, the only hit one that displays statistical significance in negative correlation with EYA4 turned out to be TRIM69 (*r* = -0.585, *P* = 0.045, Figure 1C and Supplemental Figure 1E). In sharp contrast, no correlation of other TRIM homologues, such as TRIM25, TRIM13 or TRIM37 with EYA4 was detected in this screen. We did not observe any statistically significant correlation of TRIM69 with EYA1, EYA2 and EYA3 as well, respectively (Supplemental Figure 1F-H). We thus focused on TRIM69 in subsequent analyses.

To further confirm and extend the findings observed in CCLE, we followed a secondary round investigation of the correlation between TRIM69 and EYA4 using *in silico* studies. Multi-dimensional datasets revealed that TRIM69 had mutually exclusive genomic deletion patterns with EYA4 in in many solid cancers including PDAC (Figure 1D and Supplemental Figure 1I-M). We further corroborated these observations by two independent PDAC cohorts: 1) cohort 1 contained 10 freshly collected, snap-frozen specimens from PDAC patients who had experienced radical resection, in which the relationship between TRIM69 and EYA4 was assessed by western-blotting (WB); 2) cohort 2 included 115 paraffin-embedded samples from clinical patients diagnosed with PDAC, in which TRIM69 staining intensity was determined by immunohistochemistry (IHC), and its correlation with EYA4 and other clinical characteristics was also analyzed. Notably and consistent with the results described for mutually exclusive genomic patterns between TRIM69 and EYA4, WB analyses of the PDAC specimens from cohort 1 showed that the levels of TRIM69 protein expression were negatively associated with EYA4 (*r* = -0.689, *P* = 0.027; Figure 1E). In cohort 2, 76 cases exhibited strong expression of TRIM69 and 39 cases exhibited weak expression of TRIM69, indicating that TRIM69 expression is amplified in majority of patients with PDAC. Of note, positive EYA4 was detected in 29.7% specimens with strong expression of TRIM69 and in 70.3% specimens with weak expression of TRIM69, while negative EYA4 was detected in 64.1% specimens with strong expression of TRIM69 and 35.9% specimens with weak expression of TRIM69, respectively (*r* = -0.207, *P* = 0.034; Figure 1F). Integrated analysis of gene expression profiles from the GFP-EYA4-expressing S-1 transfectants identified 158 and 116 genes that were downregulated and upregulated by EYA4, respectively 7. They were individually termed as the EYA4-deactivated or EYA4-activated gene set (Figure 1G); here the former positively, while the latter inversely correlated with TRIM69 in a prior published gene signature of PDAC as measured by gene set enrichment analysis (GSEA) (Figure 1H). These results together suggest that TRIM69 might be a mutually exclusive E3 ligase of EYA4 in human PDAC.

### TRIM69 elicits polyubiquitylation and turnover of EYA4

**Figure 2:**
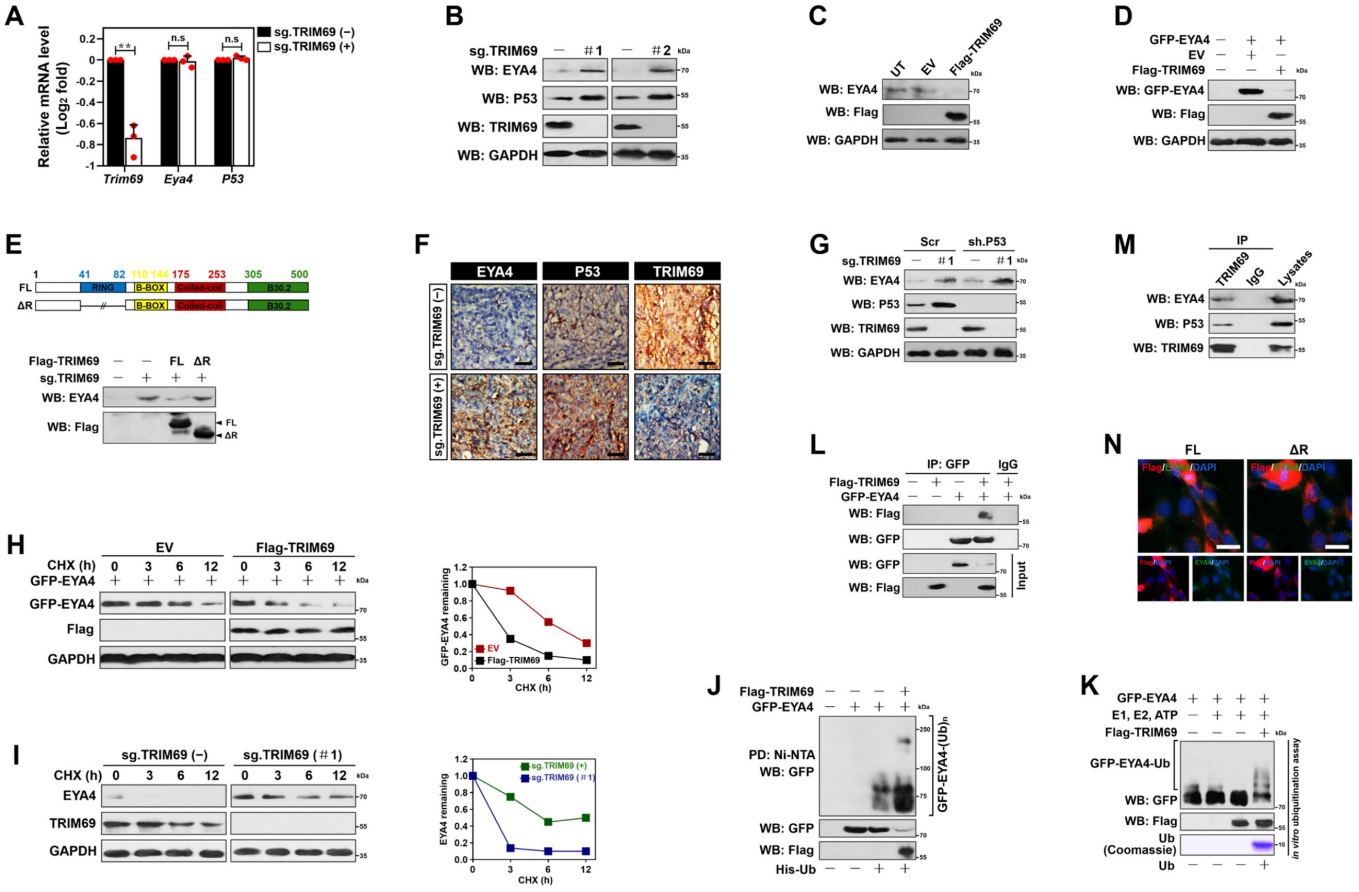
** TRIM69 elicits polyubiquitylation and turnover of EYA4. (A)** RT-qPCR analyses of EYA4 and P53 mRNA expression in SW-1990 cells with or without sg.TRIM69 transduction. Experiments were performed three times, each with quantitative RT-PCR in technical duplicate and real-time values were normalized to glyceraldehyde 3-phosphate dehydrogenase (GAPDH). Data are expressed as mean ± s.d. **P<0.01. Two-sided Student's t test was used to calculate the *P* value. n.s, no significant.** (B)** Western-blotting analyses evaluating abundance of EYA4 and P53 protein in SW-1990 cells with or without sg.TRIM69 transduction.** (C)** Western-blotting analyses comparing the levels of endogenous EYA4 expression in SW-1990 cells with or without Flag-tagged wild-type TRIM69 transfection. UT, untransfection; EV, empty vector. **(D)** Western-blotting analyses detecting the levels of EYA4 protein in GFP-EYA4-expressed SW-1990 cells with or without Flag-tagged wild-type TRIM69 transfection. **(E)** Western-blotting analyses comparing expression levels of endogenous EYA4 protein in sg.TRIM69-transduced SW-1990 cells with the Flag-tagged full-length (FL) or RING finger domain truncation (ΔR) TRIM69 reconstitution. The top panel indicates secondary structures of the entire and RING finger domain-truncated human TRIM69 protein. **(F)** Representative immunochemical images for EYA4 staining in subcutaneous xenografts formed by the indicated SW-1990 cells from nude mice at day 21 after inoculation. Scale bar = 100 μm. **(G)** Western-blotting analyses testing the levels of endogenous EYA4 and P53 protein in sg.TRIM69-transduced SW-1990 cells with scrambled shRNA (Scr) or P53 shRNA (sh.P53) transfection. **(H)** Cycloheximide (CHX) pulse-chase experiments examining turnover of EYA4 protein in GFP-EYA4-expressed SW-1990 cells with or without Flag-tagged wild-type TRIM69 transfection in the presence or absence of 20 μg/mL CHX treatment for the indicated times, respectively. **(I)** Cycloheximide (CHX) pulse-chase experiments determining turnover of endogenous EYA4 protein in SW-1990 cells with or without sg.TRIM69 transduction in the presence or absence of 20 μg/mL CHX treatment for the indicated times, respectively.** (J)** Cellular ubiquitination assays assessing polyubiquitylation levels of EYA4 in GFP-EYA4-expressed SW-1990 cells with or without Flag-tagged wild-type TRIM69 transfection, respectively. **(K)** *In vitro* ubiquitination assay of the reaction products by mixing the purified Flag-TRIM69 with E1, E2, Ub and GFP-EYA4 at 37 °C for 2 h. **(L)** Coimmunoprecipitation assay detecting the interaction between TRIM69 and EYA4 in SW-1990 cells coexpressed Flag-tagged wild-type TRIM69 and GFP-EYA4. **(M)** Coimmunoprecipitation assay determining the interaction of endogenous TRIM69 with EYA4 and P53 in SW-1990 cells. (N) Representative immunfluorescence images showing subcellular distribution and EYA4 assembly of full-length (FL) or RING finger domain truncation (ΔR) TRIM69 in SW-1990 cells. Scale bar = 25 μm.

The mutually exclusive interaction of TRIM69 with EYA4 spurred us to explore the functional role of TRIM69 on EYA4. For this purpose, we transduced S-1 cells with CRISPR-Cas9 guide RNA targeting TRIM69 (sg.TRIM69) or engineered them to express vector bearing Flag-tagged wild-type TRIM69 (Flag-TRIM69). Sg.TRIM69 transduction largely downregulated mRNA and protein expression of TRIM69, which were accompanied by the higher steady-state levels of EYA4 protein without appreciable alteration of its mRNA abundance (Figure 2A and 2B). The comparable results were yielded in S-1 cells that were transfected with short hairpin RNA (sh.RNA) and small interfering RNA (si.RNA) duplexes targeting TRIM69 (Supplemental Figure 2A-D), respectively. The siRNA-mediated knockdown (KD) of TRIM13 or TRIM37 increased EYA4 protein levels less efficiently than did of TRIM69, while TRIM25 KD had minimal effect (Supplemental Figure 2E-I). TRIM37 KD, but not TRIM13 or TRIM25 KD, modestly upregulated mRNA abundance of EYA4, which may account for the elevation of protein abundance observed in WB analyses (Supplemental Figure 2J). TRIM69 KD did not further upregulate EYA4 in cells where TRIM13 had been silenced (Supplemental Figure 2K). By contrast, ectopic TRIM69 expression greatly increased the amount of TRIM69 protein to an extent corresponding to the detectable reductions in both endogenous and exogenous EYA4 protein when compared with empty vector (EV) (Figure 2C and D). The reduction of exogenous EYA4 following TRIM69 overexpression did not occur upon bortezomib or MG132 treatment, suggesting that EYA4 is targeted for proteasomal degradation by TRIM69 (Supplemental Figure 2L). The wild-type full-length TRIM69 (TRIM69^FL^), but not the N-terminal RING finger domain truncation (TRIM69^ΔR^), abrogated the elevation of EYA4 protein caused by sg.TRIM69 transduction (Figure 2E), ruling out the possibility of an off-target impact. To test whether the degradation of EYA4 by TRIM69 could be recapitulated *in vivo*, we subcutaneously injected the sg.TRIM69-transduced S-1 cells into right flank of nude mice. We found that xenografts arose in mice inoculated with the cells expressing sg.TRIM69 contained much higher proportion of EYA4-positive staining than those in mice inoculated with the parental cells (Figure 2F). These data collectively demonstrate that TRIM69 posttranslationally downregulates EYA4 in PDAC.

TRIM69 is known to catalyze P53 turnover 14. We thus asked whether TRIM69 posttranslationally downregulates EYA4 through repressing P53. Treatment with RITA, a small-molecule P53 activator 28, efficiently activated P53 as reflected by the robust upregulation in mRNA and protein expression of two P53 target genes *Puma* and *p27^Cdkn1b^* following administration. Nevertheless, the same dosage of RITA barely influenced the abundance of EYA4 mRNA and protein (Supplemental Figure 3A and B). The sg.TRIM69-transduced cells had increased protein expression of P53, but their EYA4 protein levels were still elevated when P53 had been deleted from cell lysates (Figure 2G and Supplemental Figure 3C). The amplitude of P53 protein elevated by sg.TRIM69 in EYA4-deficient cells were similar to those in EYA4-proficient cells (Supplemental Figure 3D), implying that TRIM69 degrades EYA4 and P53 independent of each other.

We next enrolled cycloheximide pulse-chase experiments to elucidate whether TRIM69 degrades EYA4 protein by regulating its stability. To approach this, S-1 transfectants stably expressing GFP-EYA4 were exposed to cycloheximide (CHX) for various times following TRIM69 introduction, and the half-life of GFP-EYA4 protein was monitored. The half-life of GFP-EYA4 protein in cells with TRIM69 introduction was much shorter than that in cells without upon CHX treatment (Figure 2H). In stark contrast to this observation, the sg.RNA-mediated knockout (KO) of TRIM69 readily increased half-life of endogenous EYA4 protein under the same conditions (Figure 2I and Supplemental Figure 3E), validating that TRIM69 accelerates turnover of EYA4 protein. In echoing this notion, cellular ubiquitination assay of the His-tagged Ub protein immobilized on Ni^2+^-nitrilotriacetic acid (Ni-NTA)-sepharose beads with an anti-GFP antibody depicted that TRIM69 increased polyubiquitination of EYA4 (Figure 2J). TRIM69 KO, however, diminished EYA4 polyubiquitylation that could be rescued by TRIM69^FL^ but not TRIM69^ΔR^ (Supplemental Figure 3F). To clarify the direct activity of TRIM69 E3 ligase toward EYA4, we utilized a cell-free system, in which purified GFP-EYA4 protein was incubated with Flag-TRIM69 in the presence of E1, E2 and adenosine triphosphate (ATP). We found that TRIM69 substantially attached polyubiquitin chains to EYA4 (Figure 2K). TRIM69 tended to elicit the K48-linked rather than the K63-linked polyubiquitylation of EYA4 since mutation of K48 but not that of K63 residue to arginine (R) on polyubiquitin chains blocked their capture by EYA4 after TRIM69 introduction (Supplemental Figure 3G). These data propose that TRIM69 targets EYA4 protein for turnover by attaching the K48-linked polyubiquitin chains.

Examination of GFP protein immunoprecipitated from S-1 transfectants coexpressing GFP-EYA4 plus Flag-TRIM69 with an antibody against Flag identified a detectable interaction between EYA4 and TRIM69 (Figure 2L). Interaction of TRIM69 with EYA4 was also observed in a reciprocal coimmunoprecipitation assay of Flag-TRIM69 using the anti-GFP antibody (Supplemental Figure 3H). Western-blotting analysis of TRIM69 from cytoplasmic extractions with antibodies against EYA4 and P53 demonstrated that EYA4 bound to TRIM69 at endogenous levels as efficiently as did P53 (Figure 2M). The TRIM69^ΔR^ showed subcellular distribution and EYA4 assembly to a similar degree as TRIM69^FL^ did (Figure 2N), indicating that RING finger domain of TRIM69 is dispensable for its physical interaction with EYA4.

### TRIM69 is upregulated in PDAC and predicts unfavorable prognosis

**Figure 3:**
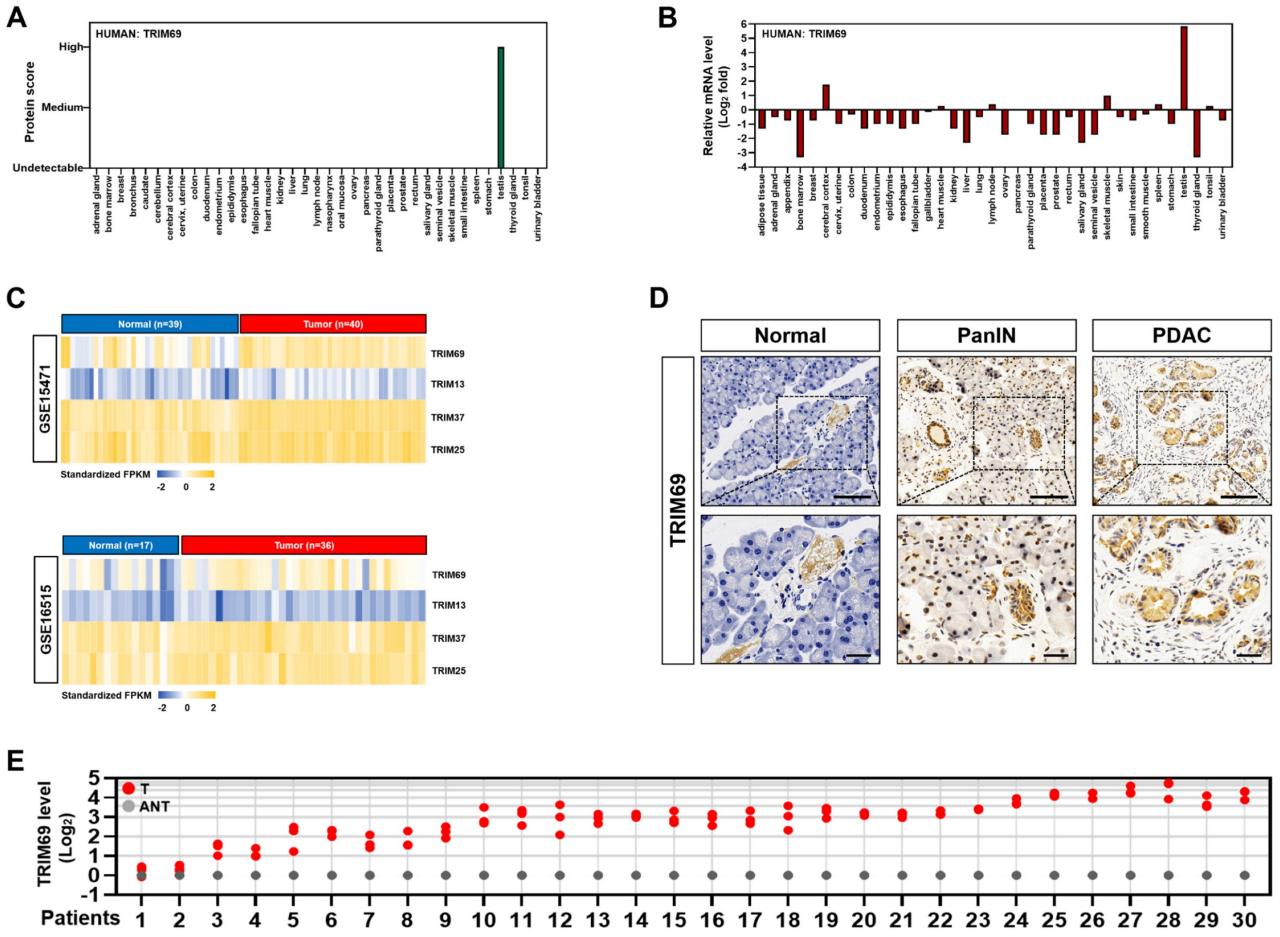
** TRIM69 is upregulated in PDAC. (A)** Protein score of human TRIM69 expression in the indicated tissues from The Human Protein Atlas. **(B)** Normalized expression of human TRIM69 mRNA in the indicated tissues from The Human Protein Atlas. **(C)** Microarray analyses of two publically available human pancreatic ductal adenocarcinoma (PDAC) studies (GSE15471 and GSE16515) from the NCBI GEO for *Trim69, Trim13, Trim37* and* Trim25* genes. **(D)** Representative immunohistochemical pictures showing TRIM69 expression from normal pancreas to PanINs and PDAC of *Pdx1*-Cre; LSL-Kras^G12D/+^; Trp53^fl/+^ (KPC) mice. Scale bar: 100 and 50 µm.** (E)** RT-qPCR analyses of *Trim69* mRNA expression in 30 PDAC tissues of patients and their matched adjacent non-tumoral tissues. Experiments were performed three times, each with quantitative RT-PCR in technical duplicate and real-time values were normalized to glyceraldehyde 3-phosphate dehydrogenase (GAPDH).

Next, we sought to pursue the clinical relevance of TRIM69 with PDAC. To this end, we first examined the expression pattern of TRIM69 protein and mRNA in 37 organs from The Human Protein Atlas and found that TRIM69 protein rather than its mRNA was completely restricted to expression in testis with no detectable expression in any other organs (Figure 3A and B). The pan-cancer survey of distinct tumor types from patients revealed that TRIM69 was recurrently upregulated in nearly all tumors including pancreatic adenocarcinoma (PAAD), which also contained much higher levels of *Ctnnb1* and *Id2* than the normal controls (Supplemental Figure 4A). Further analysis of RNA sequencing data from The Cancer Genome Atlas (TCGA) for 179 PAAD samples and 171 normal pancreas samples interrogated that TRIM69 was upregulated in PAAD tissues, in concert with the upregulation of *Ctnnb1* and *Id2* (Supplemental Figure 4B). These results coincided with the microarray datasets from three large-scale studies deposited in Oncomine database showing that the patient-derived PDAC samples had a significantly elevated TRIM69 levels when compared with normal pancreatic samples (Supplemental Figure 4C). A similar upregulation of TRIM69 was also observed in two PDAC cohorts from NCBI's Gene Expression Omnibus (GEO) database, in which GSE15471 contained 40 tumor tissues and 39 normal pancreatic tissues, and GSE16515 included 36 tumor tissues and 17 normal pancreatic tissues (Figure 3C). To further determine the expression pattern of TRIM69 protein in PDAC, we performed immunohistochemical staining in a preclinical PDAC model of *Pdx1*-Cre; LSL-Kras^G12D/+^; Trp53^fl/+^ (KPC) mice and observed that TRIM69 immunoreactivity was stepwisely augmented from normal pancreas to PanINs and PDAC (Figure 3D). Additional evidence that TRIM69 is upregulated in PDAC was reinforced by the retrospective study of our previously published data from 30 PDAC tissues of patients and their matched adjacent non-tumoral tissues (ANT) in which RNA samples were available, showing that TRIM69 was universally overexpressed in PDAC tissues as compared to non-tumoral pancreas tissues (Figure 3E).

**Figure 4:**
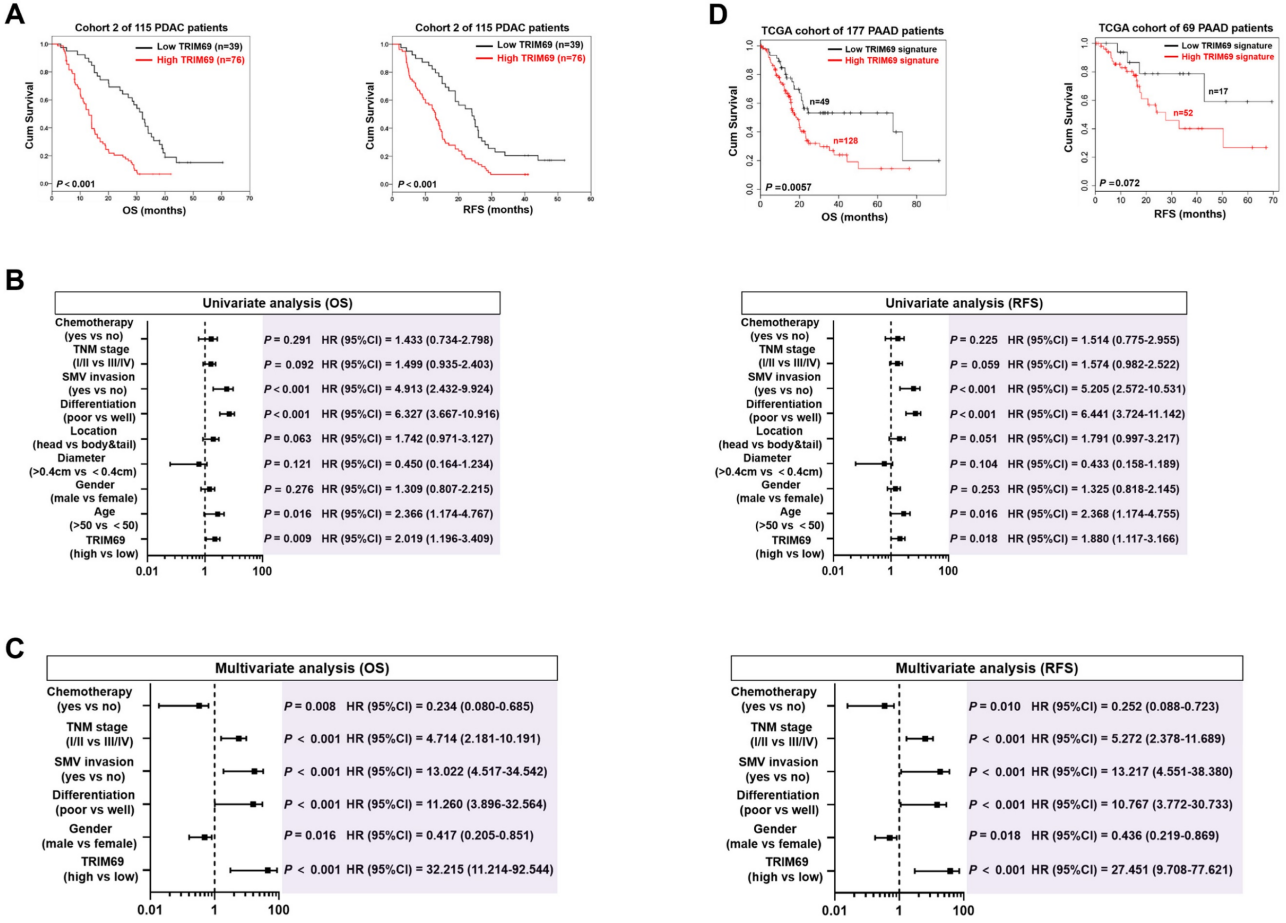
** Upregulation of TRIM69 in human PDAC predicts unfavorable prognosis. (A)** Kaplan-Meier survival curves of overall survival (OS) and recurrence-free survival (RFS) duration based on TRIM69 expression in 115 PDAC patients from Cohort 2. Log-rank test was used to calculate the *P* value. **(B)** Univariate analysis of different prognostic parameters for overall survival (OS) and recurrence-free survival (RFS) duration of 115 PDAC patients in Cohort 2. **(C)** Multivariate analysis of different prognostic parameters for overall survival (OS) and recurrence-free survival (RFS) duration of 115 PDAC patients in Cohort 2. **(D)** Kaplan-Meier curves comparing the overall survival (OS) and recurrence free survival (RFS) in the indicated pancreatic adenocarcinoma (PAAD) patients from The Cancer Genome Atlas database with low and high TRIM69 signature. Log-rank test was used to calculate the *P* value.

Given the aberrant TRIM69 enrichment in human and mouse PDAC, we tested the relationship between TRIM69 and clinicopathological characteristics of PDAC patients. To approach this, patients with both above- and below-median TRIM69 expression in cohort 2 were followed up. Correlation analysis revealed that TRIM69 statistically associated with multiple malignant PDAC characteristics (Supplemental Figure 4D), and patients with above-median TRIM69 expression displayed an inferior overall survival (OS) and recurrence-free survival (RFS) in comparison to those with below-median TRIM69 expression, quantitatively corresponding to the median times 13.05 ± 1.11 months versus 32.00 ± 2.37 months for OS and 13.00 ± 1.34 months versus 24.06 ± 3.12 months for RFS (Figure 4A), respectively. Univariate and multivariate Cox proportional hazards analysis revealed that TRIM69 was an independent prognostic marker of PDAC aggressiveness with significant hazard ratios for predicting clinical outcome (Figure 4B and C). Kaplan-Meier survival curves generated by gene sets which are positively correlated with TRIM69 (defined as “TRIM69 signature”) using TCGA datasets also showed that increased expression of TRIM69 signature predicted worse patient survival (Figure 4D). Thus, TRIM69 is physiologically restricted to testis but frequently amplified in PDAC, and its expression predicts poor prognosis.

### TRIM69 impedes the tumor-suppressive phenotypes of EYA4

**Figure 5:**
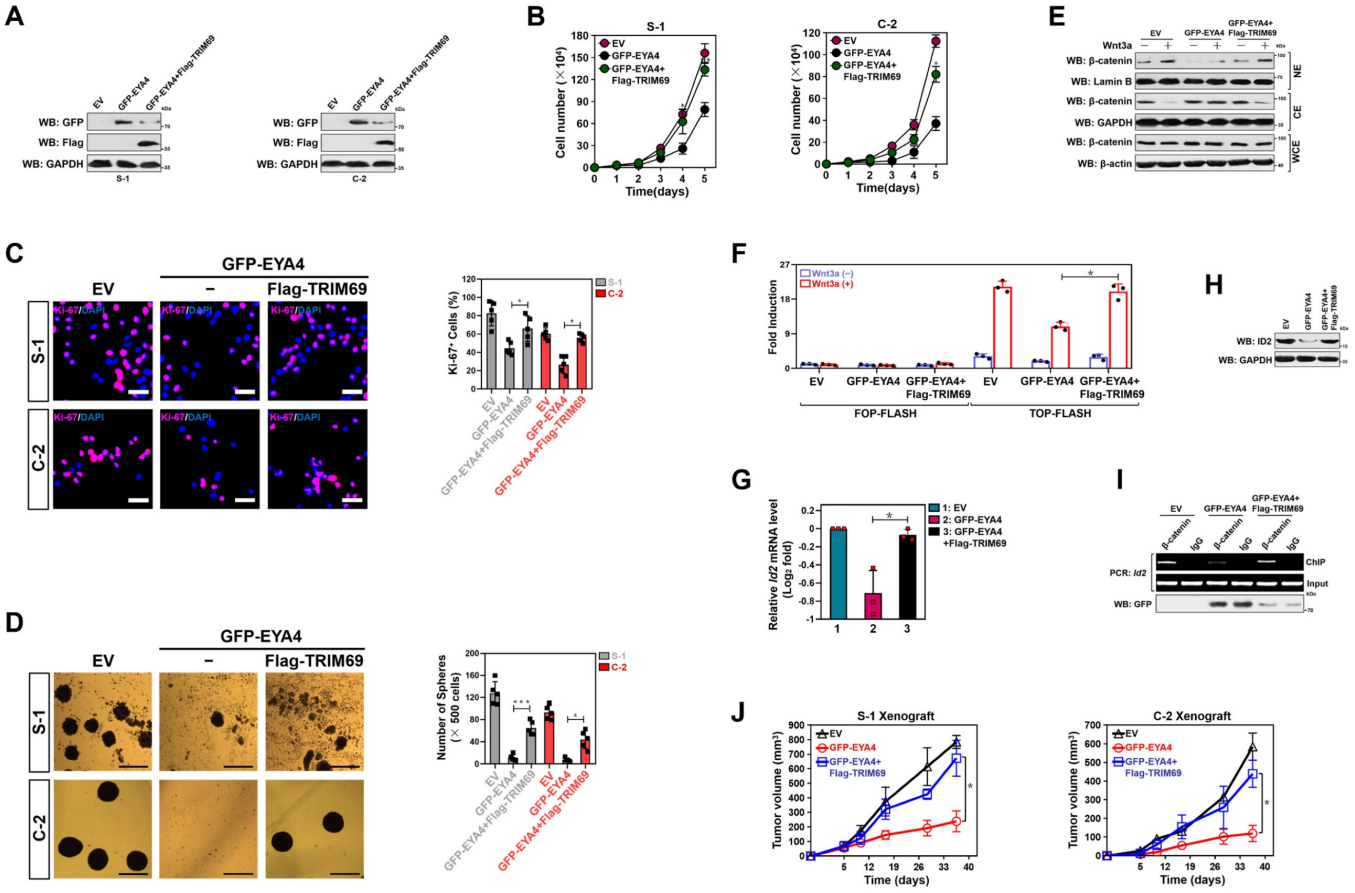
** TRIM69 impedes the tumor-suppressive phenotypes of EYA4. (A)** Western-blotting analyses examining GFP and Flag protein expression in SW-1990 (S-1) or Capan-2 (C-2) cells with GFP-EYA4 transfection or GFP-EYA4 plus Flag-tagged wild-type TRIM69 cotransfection. **(B)** Trypan blue assay measuring cell growth of SW-1990 (S-1) and Capan-2 (C-2) cells with GFP-EYA4 transfection or GFP-EYA4 plus Flag-tagged wild-type TRIM69 cotransfection. Data are expressed as mean ± s.d. of three independent experiments. *P<0.05 and **P<0.01. Two-sided ANOVA with Bonferroni post hoc t test correction was used to calculate the *P* value. EV, empty vector. **(C)** Representative immunfluorescence images and quantification of Ki-67 staining in SW-1990 (S-1) and Capan-2 (C-2) cells with GFP-EYA4 transfection or GFP-EYA4 plus Flag-tagged wild-type TRIM69 cotransfection. Data are expressed as mean ± s.d. of five independent experiments. *P<0.05. Two-sided ANOVA with Bonferroni post hoc t test correction was used to calculate the *P* value. Scale bar = 50 μm. **(D)** Representative images and quantification of sphere formation in SW-1990 (S-1) and Capan-2 (C-2) cells with GFP-EYA4 transfection or GFP-EYA4 plus Flag-tagged wild-type TRIM69 cotransfection. Data are expressed as mean ± s.d. of five independent experiments. *P<0.05 and ***P<0.001. Two-sided ANOVA with Bonferroni post hoc t test correction was used to calculate the *P* value. Scale bar = 200 μm. **(E)** Subcellular fractionation analyses examining accumulation of nuclear and cytoplasmic β-catenin protein in SW-1990 cells with GFP-EYA4 transfection or GFP-EYA4 plus Flag-tagged wild-type TRIM69 cotransfection in the presence or absence of Wnt-3a (20 ng/mL) stimulation. GAPDH and Lamin B were used as internal control of cytoplasmic and nuclear extractions, respectively. NE, nuclear extraction; CE, cytoplasmic extraction; WCE, whole cell extraction. **(F)** Dual-luciferase reporter assays of TCF/LEF transcriptional activity in SW-1990 cells with GFP-EYA4 transfection or GFP-EYA4 plus Flag-tagged wild-type TRIM69 cotransfection in the presence or absence of Wnt-3a (20 ng/mL) stimulation. Data are expressed as mean ± s.d. of three independent experiments. *P<0.05. Two-sided ANOVA with Bonferroni post hoc t test correction was used to calculate the *P* value. **(G)** RT-qPCR analyses of *Id2* gene expression in SW-1990 cells with GFP-EYA4 transfection or GFP-EYA4 plus Flag-tagged wild-type TRIM69 cotransfection in the presence of Wnt-3a (20 ng/mL) stimulation. Experiments were performed three times, each with quantitative RT-PCR in technical duplicate and real-time values were normalized to glyceraldehyde 3-phosphate dehydrogenase (GAPDH). Data are expressed as mean ± s.d. *P<0.05. Two-sided ANOVA with Bonferroni post hoc t test correction was used to calculate the *P* value. **(H)** Western-blotting analyses determining ID2 protein expression in SW-1990 cells with GFP-EYA4 transfection or GFP-EYA4 plus Flag-tagged wild-type TRIM69 cotransfection in the presence of Wnt-3a (20 ng/mL) stimulation. **(I)** ChIP analysis for β-catenin binding to *Id2* gene promoter in SW-1990 cells with GFP-EYA4 transfection or GFP-EYA4 plus Flag-tagged wild-type TRIM69 cotransfection in the presence of Wnt-3a (20 ng/mL) stimulation using the indicated antibodies. **(J)** Tumor volume of xenografts excised from the tumor-bearing mice. The indicated SW-1990 or Capan-2 cells (1×10^7^) were subcutaneously inoculated into the right flank of nude mice. The mice were sacrificed and the tumors were excised and measured on day 35. Data are presented as mean ± s.d. *P<0.05. Two-sided ANOVA with Bonferroni post hoc t test correction was used to calculate the *P* value.

Since TRIM69 elicits polyubiquitylation and turnover of EYA4, it is plausible that tumor-suppressive roles of EYA4 could be blocked by TRIM69. As hypothesized, trypan blue assay showed that S-1 transfectants had impaired growing capacities as early as three days after stable GFP-EYA4 expression, with average 2-fold less metabolically active cells than the EV controls at 5 days. Introduction of Flag-TRIM69, however, led to a reversal of this growing defect (Figure 5A and B). Forced EYA4 expression in S-1 cells reduced the number of Ki-67 staining, an index of cell proliferation, while such effect was substantially abrogated by concurrent expression of TRIM69 (Figure 5C). The reverse effects also manifested by PDAC self-renewal experiments in a three-dimensional culture system. The GFP-EYA4-expressing S-1 cells had compromised generation and volume of sphere during the testing frame time in contrast to their EV counterparts; whereas the GFP-EYA4 plus Flag-TRIM69-coexpressing cells did so to a much lesser extent (Figure 5D). Similar results were yielded in C-2 cells (Figure 5A-5D), suggesting that TRIM69 abrogates the EYA4-driven growth inhibition.

EYA4 exerts its tumor-suppressive functions mainly through deactivation of β-catenin/inhibitor of DNA binding 2 (ID2) cascade^7^, raising the possibility that TRIM69 might thwart the EYA4-driven deactivation of β-catenin/ID2 cascade. Indeed, overexpressing EYA4, like silencing cAMP-dependent protein kinase (PKA), remarkably impaired phosphorylation of β-catenin Ser675 that could be mitigated by TRIM69 (Supplemental Figure 5A). In accordance with the result described for β-catenin phosphorylation, TRIM69 attenuated the EYA4-inducible nuclear export of β-catenin, as judged by the increased nuclear β-catenin accumulation in the EYA4 plus TRIM69-coexpressing cells as compared with the EYA4-expressing cells upon Wnt3a stimuli from the subcellular fractionation (Figure 5E) and immunofluorescence analyses (Supplemental Figure 5B), respectively. To surmise whether EYA4 represses β-catenin transactivation in a TRIM69-dependent fashion, we transiently transfected a luciferase reporter containing either the wild-type or mutant transcription factor (TCF)/lymphoid enhancer binding factor (LEF) DNA binding sequence into the GFP-EYA4-expressing or the GFP-EYA4 plus Flag-TRIM69-coexpressing cells and examined the resultant effects. As shown in Figure 5F, a profound decline in the transcriptional activity of TCF/LEF was observed following EYA4 single expression instead of EYA4 plus TRIM69 coexpression. TRIM69 reversed the mRNA and protein levels of ID2 downregulated by EYA4 overexpression (Figure 5G and H), presumably due to the incomplete restoration of β-catenin recruitment at *Id2* gene promoter (Figure 5I). These data collectively implicate that TRIM69 prevents the EYA4-driven deactivation of β-catenin/ID2 signalling in PDAC cells.

We next validated the growth-inhibitory defects using PDAC xenografts. Although the S-1 or C-2 transfectants stably expressing EYA4 were still tumorigenic *in vivo*, both the tumor growth rate and mass from mice inoculated with these transfectants were much slower and smaller than those in mice that were inoculated with the transfectants expressing EV or transfectants coexpressing EYA4 plus TRIM69 (Figure 5J and Supplemental Figure 5C and D), respectively. Histological analyses using immunofluorescence (IF) further revealed that the xenografts derived from transfectants coexpressing EYA4 plus TRIM69, rather than those derived from transfectants expressing EYA4, had comparable percentage of Ki-67 staining to the xenografts derived from transfectants expressing EV controls (Supplemental Figure 5E). Thus, TRIM69 impedes tumor-suppressive phenotypes of EYA4 *in vivo*.

### Targeting TRIM69 preferentially induces lethality in EYA4-deficient PDAC cells

**Figure 6:**
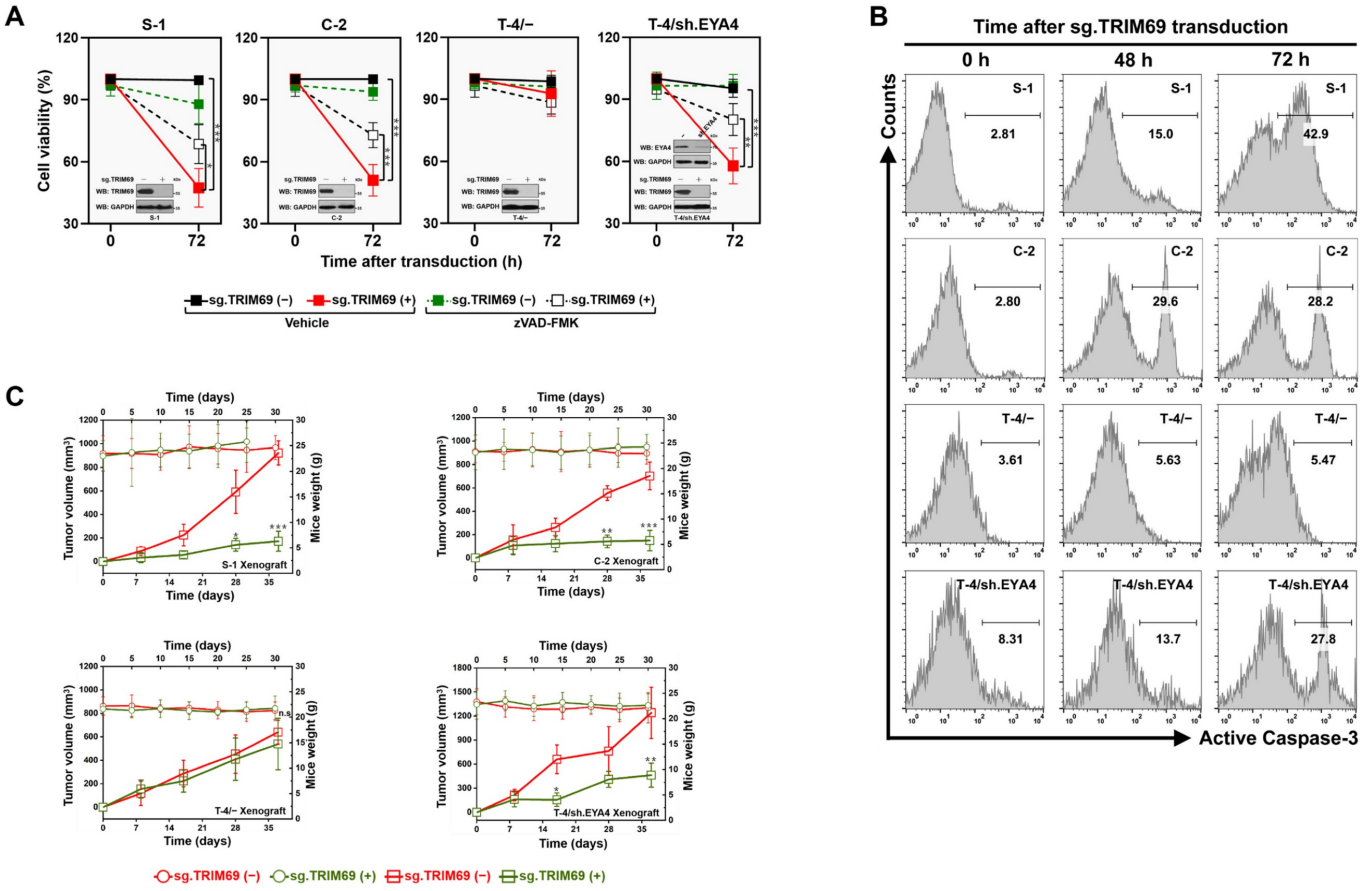
** Targeting TRIM69 preferentially induces lethality in EYA4-deficient PDAC cells. (A)** MTT assays measuring cell viability of SW-1990 (S-1) or Capan-2 (C-2), T3M-4 (T-4/-) as well as EYA4 shRNA (sh.EYA4)-expressed T3M-4 (T-4/sh.EYA4) cells with or without sg.TRIM69 transduction in the presence or absence of zVAD-FMK (20 μM) pretreatment, respectively. Inset: Western-blotting analyses comparing levels of TRIM69 protein in SW-1990 (S-1), Capan-2 (C-2) and T3M-4 (T-4) cells with sg.TRIM69 transduction, and detecting expression of EYA4 protein in T3M-4 (T-4) cells with or without EYA4 shRNA (sh.EYA4) transfection, respectively. Data are presented as mean ± s.d. *P<0.05, **P<0.01 and ***P<0.001. Two-sided ANOVA with Bonferroni post hoc t test correction was used to calculate the *P* value. **(B)** Histogram of flow cytometry for detecting caspase-3 activation in SW-1990 (S-1) or Capan-2 (C-2), T3M-4 (T-4/-) as well as EYA4 shRNA (sh.EYA4)-expressed T3M-4 (T-4/sh.EYA4) cells with or without sg.TRIM69 transduction at the indicated times, respectively. **(C)** Tumor volume of subcutaneous xenografts formed by SW-1990 (S-1) or Capan-2 (C-2), T3M-4 (T-4/-) as well as EYA4 shRNA (sh.EYA4)-expressed T3M-4 (T-4/sh.EYA4) cells with or without sg.TRIM69 transduction from nude mice at the indicated times. Inset: body weight of the tumor-bearing mice. Data are presented as mean ± s.d. *P<0.05, **P<0.01 and ***P<0.001 versus sg.TRIM69 (-). Two-sided ANOVA with Bonferroni post hoc t test correction was used to calculate the *P* value.

Considering the mutually exclusive interaction between TRIM69 and EYA4, we examined the therapeutic potential of targeting TRIM69 in the EYA4-deficient PDAC cells. To address this issue, cell viability of S-1 and C-2 cells were measured after sg.TRIM69 transduction. Sg.TRIM69 abolished TRIM69 expression and dramatically increased cell death. However, identical transduction of sg.TRIM69 had little effect on the EYA4-proficient T-4 cells. Silencing EYA4 with shRNA in T-4 cells sensitized them to the sg.TRIM69-induced death (Figure 6A). Likewise, depletion of TRIM69 by either siRNA or shRNA duplexes led to death of S-1 and C-2 cells, while T-4 cells became susceptible to the TRIM69 depletion-inducible death only after silencing EYA4 (Supplemental Figure 6A-D). Pretreatment with benzyloxycarbonyl-Val-Ala-Asp-fluoromethylketone (zVAD-FMK), a pan caspase inhibitor, alleviated the sg.TRIM69-inducible death in S-1, C-2 cells and the EYA4-silenced T-4 cells (Figure 6A). To determine if the augmented sensitivity to TRIM69 defect was a repercussion from active caspase, we enrolled flow cytometry for detecting caspase-3 activation and found that S-1 and C-2 cells, but not T-4 cells, with sg.TRIM69 transduction had higher caspase-3 activity than those without, whereas sg.TRIM69 substantially elevated caspase-3 activity of T-4 cells in the absence of EYA4 (Figure 6B). These data demonstrate that targeting TRIM69 results in lethality in the EYA4-deficient PDAC cells through modulation of caspase activity.

To interrogate whether targeting TRIM69 could impair tumorigenicity *in vivo*, we monitored subcutaneous xenografts in nude mice. Genetic knockout (KO) of TRIM69 significantly inhibited tumor development, as evidenced by the decreased growth rate, weight and size of tumors from mice inoculated with the sg.TRIM69-transduced S-1 and C-2 cells in comparison to those from mice that were inoculated with the sg.TRIM69-nontransduced cells, respectively (Figure 6C and Supplemental Figure 6E and F). Deletion of EYA4 in T-4 cells using shRNA was sufficient to render the growth-inhibitory effect of TRIM69 KO, which by itself had no effect on T-4 tumor growth in mice (Figure 6C and Supplemental Figure 6G and H). In all cases, however, there were no significant differences in body weight between mice bearing TRIM69 KO tumors and those bearing TRIM69-intact tumors. Taken together, our results highlight the importance of targeting TRIM69 as a feasible strategy for the EYA4-deficient PDAC.

### ERK2-dependent phosphorylation of EYA4 at Ser37 is instrumental for the TRIM69-elicited EYA4 polyubiquitylation and turnover

**Figure 7:**
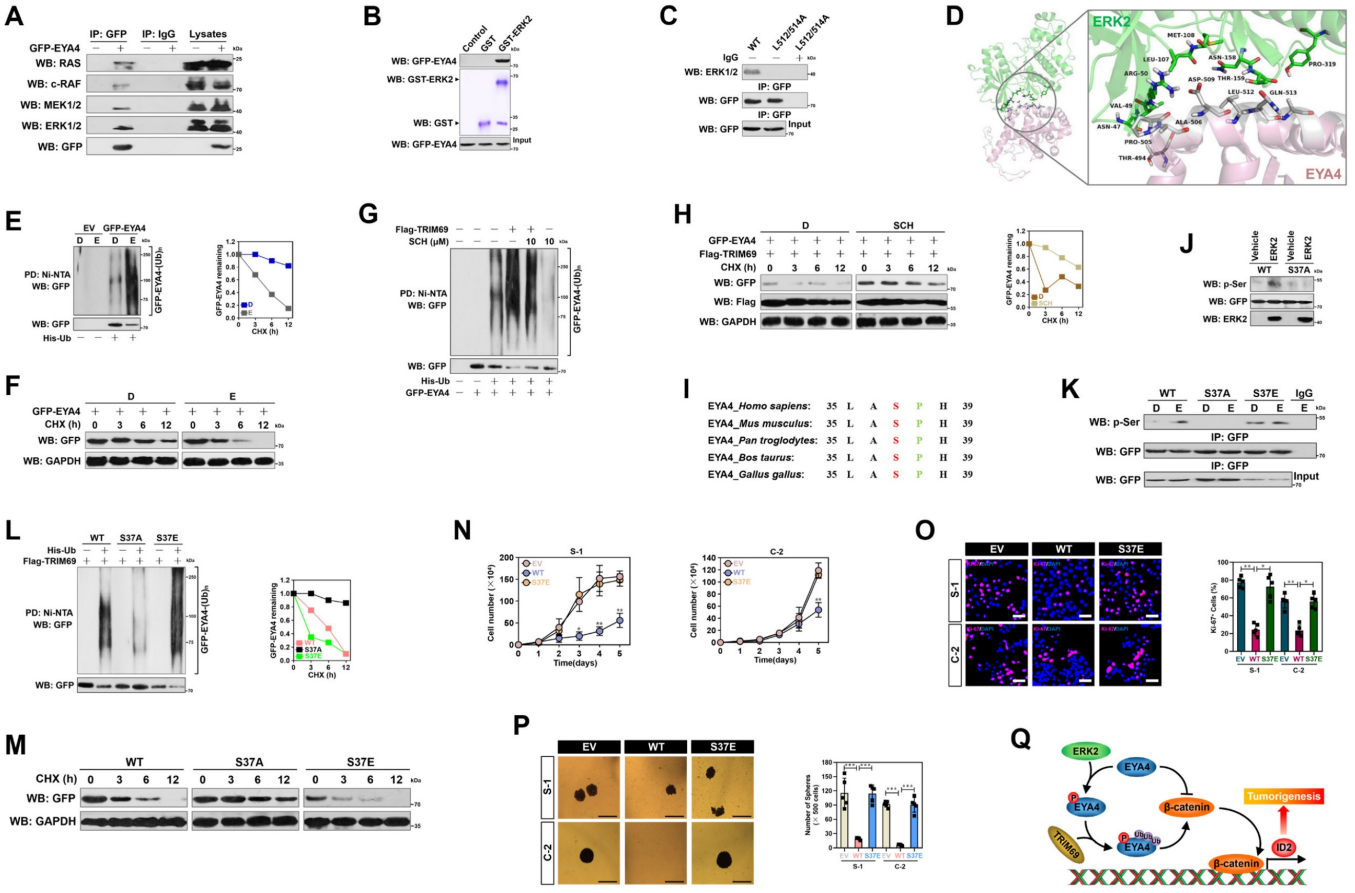
** ERK2-dependent phosphorylation of EYA4 at Ser37 is instrumental for the TRIM69-elicited EYA4 polyubiquitylation and turnover. (A)** Co-immunoprecipitation assay examining the interaction between EYA4 and RAS, c-RAF, MEK1/2 and ERK1/2 in GFP-EYA4-expressed SW-1990 cells. **(B)** GST pull-down assay testing the interaction between recombinant GST-ERK2 protein and purified GFP-EYA4 protein from GFP-EYA4-expressed SW-1990 cells. **(C)** Coimmunoprecipitation assay detecting the interaction between ERK1/2 and GFP-tagged wild-type EYA4 or mutant EYA4 L512/514A in SW-1990 cells. **(D)** Three-dimensional structure model of ERK2-EYA4 complex. The interacting residues involved in the interface between ERK2 and D-site peptide of MAPK docking domain at EYA4 are as indicated. **(E)** Cellular ubiquitination assays evaluating polyubiquitylation levels of EYA4 in GFP-EYA4-expressed SW-1990 cells with EGF (100 ng/mL) stimulation. D, DMSO; E, EGF; PD, pull-down; Ni-NTA, Ni^2+^-nitrilotriacetic acid. **(F)** Cycloheximide (CHX) pulse-chase experiments determining the turnover of EYA4 protein in GFP-EYA4-expressed SW-1990 cells with EGF (100 ng/mL) stimulation in the presence or absence of 20 μg/mL CHX treatment for the indicated times, respectively. **(G)** Cellular ubiquitination assays assessing polyubiquitylation levels of EYA4 in GFP-EYA4-expressed SW-1990 cells with Flag-tagged wild-type TRIM69 transfection in the presence or absence of 10 μM SCH772984 (SCH) administration. D, DMSO. **(H)** Cycloheximide (CHX) pulse-chase experiments comparing the turnover of EYA4 protein in GFP-EYA4-expressed SW-1990 cells with Flag-tagged wild-type TRIM69 transfection in the presence or absence of 10 μM SCH772984 (SCH) administration. D, DMSO. **(I)** Alignment of the highly conserved Ser37/Pro38 phosphorylation motif of ERK2 within EYA4 protein from Homo sapiens to Gallus gallus. **(J)** *In vitro* protein kinase assay for detection of EYA4 Ser phosphorylation with mixing recombinant ERK2 protein and purified GFP-tagged wild-type EYA4 (WT) or mutant EYA4 Ser37A (S37A) protein followed by western-blotting analyses using an anti-phosphoserine antibody. (K) Coimmunoprecipitation assay comparing the levels of EYA4 Ser phosphorylation in SW-1990 cells transfected with GFP-tagged wild-type EYA4 (WT) or mutant EYA4 Ser37A (S37A) or Ser37E (S37E) in the presence of EGF (100 ng/mL) stimulation using an anti-phosphoserine antibody. **(L)** Cellular ubiquitination assays detecting polyubiquitylation levels of EYA4 in SW-1990 cells transfected with GFP-tagged wild-type EYA4 (WT) or mutant EYA4 Ser37A (S37A) or Ser37E (S37E) in the presence of Flag-tagged wild-type TRIM69 introduction. (M) Cycloheximide (CHX) pulse-chase experiments examining the turnover of EYA4 protein in SW-1990 cells transfected with GFP-tagged wild-type EYA4 (WT) or mutant EYA4 Ser37A (S37A) or Ser37E (S37E) in the presence or absence of 20 μg/mL CHX treatment for the indicated times, respectively. **(N)** Trypan blue assay measuring cell growth of SW-1990 (S-1) and Capan-2 (C-2) cells transfected with empty vector (EV), GFP-tagged wild-type EYA4 (WT) or mutant EYA4 Ser37E (S37E) at the indicated times. Data are expressed as mean ± s.d. of three independent experiments. *P<0.05 and **P<0.01 versus EV and S37E. Two-sided ANOVA with Bonferroni post hoc t test correction was used to calculate the *P* value. **(O)** Representative immunfluorescence images and quantification of Ki-67 staining in SW-1990 (S-1) and Capan-2 (C-2) cells transfected with empty vector (EV), GFP-tagged wild-type EYA4 (WT) or mutant EYA4 Ser37E (S37E). Data are expressed as mean ± s.d. of five independent experiments. *P<0.05 and **P<0.01. Two-sided ANOVA with Bonferroni post hoc t test correction was used to calculate the *P* value. Scale bar = 50 μm. **(P)** Representative images and quantification of sphere formation in SW-1990 (S-1) and Capan-2 (C-2) cells transfected with empty vector (EV), GFP-tagged wild-type EYA4 (WT) or mutant EYA4 Ser37E (S37E). Data are expressed as mean ± s.d. of five independent experiments. ***P<0.001. Two-sided ANOVA with Bonferroni post hoc t test correction was used to calculate the *P* value. Scale bar = 200 μm. **(Q)** Schematic diagram proposing a pivotal role for the TRIM69-elicited EYA4 polyubiquitination and turnover by the ERK2-inducible phosphorylation of EYA4 at Ser37 in directing PDAC development through activation of β-catenin/ID2 cascade.

To investigate the underlying mechanism of how TRIM69 confers EYA4 turnover, we examined the association of RAS/RAF/MEK/ERK axis with EYA4 since aberrant activation of this route accounts for more than 90% oncogenic events during PDAC initiation and progression. WB analysis of the GFP-EYA4 immobilizing in agarose beads with antibodies against RAS, c-RAF, MEK1/2 or ERK1/2 depicted that ERK1/2 interacted with EYA4 to a stronger degree than did RAS, c-RAF and MEK1/2 (Figure 7A). GFP-EYA4 was detected in immunoprecipitate (IPs) of endogenous ERK1/2 in a reciprocal co-IP assay as well (Supplemental Figure 7A). An *in vitro* glutathione S-transferase (GST) pulldown assay with mixing GST-ERK2 and purified GFP-EYA4 showed that EYA4 interacted with ERK2 (Figure 7B), which indicates a direct binding between the two proteins.

MAPKs encompass acidic common docking domain and glutamic acid aspartic acid pocket in a docking groove for substrates binding, while substrates bind to ERK through a consensus MAPK docking D-site comprised of K/R-X_1-6_-φ-X-φ motif (here X represents any amino acid [aa] and φ denotes the hydrophobic aa) 29. We found an evolutionarily conserved D-site (507-KRDAWLQL-514) in C-terminal domain of EYA4, which displayed the same patterns as other MAPK substrates did (Supplemental Figure 7B). When the hydrophobic L512/514 residues in D-site were replaced by alanines (A), EYA4 lost its ability to interact with ERK1/2 (Figure 7C), indicating that L512/514 of D-site in EYA4 is required for its interaction with ERK2 docking groove. These findings were consistent with our three-dimensional model of ERK2-EYA4 complex, where the ERK2-EYA4 interaction was anchored by hydrophobic bonds for which residues N47, V49, L107, M108, N158, T159 and P319 of ERK2 (green) pack against residues T494, P505, A506, L512 and Q513 of EYA4 (gray) or bound through salt bridges for which the charged residue R50 of ERK2 (green) packs against the charged residue D509 of EYA4 (gray) (Figure 7D). In support of this, activation of ERK1/2 by EGF stimuli or introduction of the constitutively active MEK1CA accelerated EYA4 polyubiquitylation and turnover (Figure 7E, 7F, Supplemental Figure 7C and D). Pretreatment of the ERK1/2-selective inhibitor SCH772984 (SCH) abolished ERK1/2 T202/Y204 phosphorylation and dismantled the TRIM69-elicited polyubiquitylation of EYA4 (Figure 7G, 7H and Supplemental Figure 7E). Similar results were yielded when ERK2 had been silenced from cells using RNA interference (Supplemental Figure 7F and G), implying that ERK2 is essential for the TRIM69-elicited EYA4 polyubiquitylation and turnover.

Further sequence analysis of EYA4 protein identified an ERK consensus phosphorylation motif (serine-proline) at the Ser37/Pro38 residues (Figure 7I). The proof-of-principle kinase assay *in vitro* with mixing purified GFP-EYA4 and recombinant ERK2 followed by WB analysis using an anti-phospho-serine antibody showed that ERK2 phosphorylated EYA4, but such effect did not occur when Ser37 residue was mutated into a nonphosphorylatable alanine (A) (Figure 7J). Introduction of the mutant S37A made EYA4 resistant to the EGF-stimulated Ser phosphorylation, reminiscent of either SCH pretreatment or ERK2 RNAi interference, but mutation of Ser37 into a phosphorylation-mimetic glutamine (E) allows EYA4 to undergo Ser phosphorylation even in the absence of EGF stimuli (Figure 7K and Supplemental Figure 7H and I). The S37A mutation perturbed the ability of TRIM69 to induce polyubiquitylation and turnover of EYA4, despite the phosphorylation-mimetic EYA4 S37E mutation had higher polyubiquitylation levels than wild-type EYA4 or mutant EYA4 S37A (Figure 7L and M).

Unlike wild-type EYA4, EYA4 S37E was unable to suppress cell proliferation and sphere formation in both S-1 and C-2 cells (Figure 7N-P), perhaps due to its rapid turnover rate and low stable protein levels within cellular compartment. Cells expressing EYA4 S37E had intact nuclear accumulation of β-catenin and *Id2* transcription upon Wnt-3a stimuli (Supplemental Figure 7J and K), suggesting that Ser37 autophosphorylation restrains the tumor-suppressive phenotypes of EYA4.

## Discussion

More than 85% of PDAC patients is diagnosed at advanced stages with unresectable tumors, and the conventional therapy only prolongs patients' survivals to a median of 6 months. Accordingly, an in-depth understanding of pathological characterization regarding pancreatic carcinogenesis would lay framework for developing innovative therapeutic strategy. The anti-neoplastic role of EYA4 recently gains considerable interest since epigenetic silencing of eya4 gene has been found to be associated with oncogenic signal amplification, tumor aggressiveness and unfavorable prognosis 3, 30-32. On the other hand, targeting ubiquitin-proteasome system (UPS) inhibits proliferation and induces apoptosis in cancer cells. These hints spur us to presume that certain E3 ubiquitin ligase might function as the potential intermediates to elicit EYA4 turnover, which would then contribute to initiation and progression of PDAC.

Our current study validate TRIM69 as a mutually exclusive E3 ligase targeting EYA4 for polyubiquitylation and turnover, thereby perturbing its ability to deactivate β-catenin/ID2 pathway and suppress cell proliferation, self-renewal as well as tumor development of PDAC cells. Combined analyses of data from clinical PDAC patients, KPC mice and *in silico* database demonstrate that TRIM69 expression is elevated in human and mouse PDAC, and predicts unfavorable prognosis. TRIM69 KO preferentially induces lethality *in vitro* and impairs tumorigenesis of the EYA4-deficient PDAC cells *in vivo*. Biochemical and molecular evidence further propose that the ERK2-inducible Ser37 phosphorylation is instrumental for polyubiquitylation and turnover of EYA4 elicited by TRIM69. Our future work will aim at ascertaining whether pharmacological targeting of TRIM69 with small-molecule inhibitor could effectively benefits therapeutic outcomes in the EYA4-deficient PDAC and exploring the precise mechanism responsible for the synthetic lethal interaction between TRIM69 and EYA4.

It is noteworthy that the evolutionarily conserved TRIM family regulates both oncogenic and tumor-suppressive molecules. For instance, TRIM65 facilitates ubiquitylation of trinucleotide repeat containing adaptor 6A (TNRC6A), downregulating expression of miR-138-5p and conferring the ATG7-mediated chemoresistance in non-small-cell lung cancer (NSCLC) 33. TRIM67 dampens P53 degradation, which in turn transcriptionally upregulates *Trim67*, forming a reciprocal positive feedback loop that boosts the P53-inducible cell apoptosis in colorectal cancer 34. Our study identifies EYA4 as a proteolytic substrate of TRIM69, which accelerates the K48-linked polyubiquitylation and turnover. Intriguingly, we unearth that the proteolytic role of TRIM69 in EYA4 is independent of P53. Since mutational deactivation of *P53* is a prevalent genotype in human cancers 35, our findings suggest that TRIM69 could be a therapeutic target applicable to a wide range of the EYA4-deficient PDAC regardless of their P53 status. We observe that TRIM69 evokes, through degrading EYA4, nuclear β-catenin accumulation and transcription of *Id2* gene, which had been reported to favor cancer progression via fostering mitosis, stem cell self-renewal and angiogenesis 36. The ability of TRIM69 to destabilize EYA4 and activate β-catenin/ID2 pathway might also function in connection with ERK2 kinase, which binds to D-site of substrates through its MAPK docking groove. Our study unveil D-site motif in EYA4 protein and confirm that the interaction of EYA4 with ERK2 is disrupted when the conserved residues on D-site are replaced by alanines. Despite the biological function of EYA4 D-site remains unclear, our data provide a scope toward an explanation that the D-site-mediated ERK2-EYA4 interation is essential for EYA4 Ser37 phosphorylation. Whether Ser37 phosphorylation engages in any enzyme activity of EYA4 is unknown, yet this phosphorylated event has a negative impact on EYA4 stabilization. Activated ERK2 may also promote EYA4 phosphorylation and thus render the accessibility of EYA4 to TRIM69 for turnover. Congruently, our analyses from clinical data demonstrate that TRIM69 amplification not only inversely correlates with, but also exhibits mutually exclusive genomic deletion patterns with EYA4.

Gene whose expression is restricted to cancers or immune-privileged sites such as the testes and early embryo has been historically identified as manipulator of oncogenic events, including mitotic fidelity, DNA damage repair (DDR) and tumor-immune networks 37. Our study reveals that TRIM69 protein is normally defective in somatic tissues except testes but overexpressed in PDAC. In addition to elicit EYA4 polyubiquitylation and turnover, it is plausible that TRIM69 contributes to PDAC development through additional mechanisms. Although TRIM69 transcription has not yet been reported to play a role in cancer, the presence of recurrent TRIM69 amplification involving escape from mitotic catastrophe-induced death in Kras cancer strongly suggests that TRIM69 has pro-tumorigenic capacity. Our observations show high expression of TRIM69 is significantly associated with poor survival and serves as an independent predictive biomarker in PDAC patients, which provide clinical implication for the proposal that TRIM69 may be a promising druggable target for PDAC therapy. Further systems biology and prospective studies of larger cohorts are warranted to explore the oncogenic role of TRIM69 in a holistic manner.

One way to target genetic defects in cancer is synthetic lethality. Synthetic lethality pinpoints a genetic interaction between two molecules or two pathways, in which loss of either one alone has little effect on cell survival, but simultaneous loss of both results in apoptosis 38. Our current study demonstrate that depletion of TRIM69, which has mutually exclusive patterns with EYA4, is efficient in killing the EYA4-deficient PDAC cells rather than the EYA4-proficient cells. These findings recognize TRIM69 as a potential synthetic-lethal partner of EYA4 and provide a rationale for the discovery of targetable vulnerabilities in PDAC harboring EYA4 deficiency. Intensive functional studies will be needed to verify whether TRIM69 and EYA4 act in parallel pathways that converge on the same tumorigenic process.

The current findings, together with those from previous reports 7, greatly improve our understanding of the regulatory role of upstream signaling molecules in EYA4/β-catenin/ID2 cascade during PDAC development by the following evidence. (1) PKM2 serves as a bona fide E3 ligase, eliciting polyubiquitylation and turnover of EYA4. (2) TRIM69 is upregulated and acts as an independent prognostic factor of PDAC patients. (3) TRIM69 impairs the suppressive roles of EYA4 in β-catenin/ID2 activation and PDAC tumorigenesis. The observation that TRIM69-dependent EYA4 phosphorylation and turnover mediated by ERK2, which drives β-catenin/ID2 activation, is required for PDAC cell growth and tumorigenesis and that the expression levels of TRIM69 and EYA4 correlate with PDAC prognosis may provide a molecular rationale for developing innovative therapy in PDAC harboring EYA4 deficiency/mutation.

In summary, we uncover a previously unappreciated mechanism whereby TRIM69 imparts PDAC development through eliciting polyubiquitylation and turnover of EYA4 mediated by ERK2 (Figure 7Q). Activated ERK2 leads to EYA4 phosphorylation at Ser37 and its assembly with TRIM69, which further elicits EYA4 polyubiquitylation and turnover for activation of β-catenin/ID2 cascade and PDAC development. The identification of ERK2-dependent Ser37 phosphorylation as a prerequisite for EYA4 polyubiquitylation and turnover by TRIM69 links the oncogenic Kras/MAPK/ERK signaling to UPS machinery, and suggests that approach of targeting TRIM69 may have therapeutic value in PDAC harboring EYA4 deficiency.

## Supplementary Material

Supplementary figures and table.
