# Supplementary material for: Tripartite motif containing 69 elicits ERK2-dependent EYA4 turnover to impart pancreatic tumorigenesis
Source: J Cancer. 2023 Jan 1;14(2):200–18. doi: 10.7150/jca.79905 (PMC9891873; doi:10.7150/jca.79905)
Supplement: Supplementary file 1 — Supplementary figures and table. [file jcav14p0200s1.pdf]

Supplemental Figure legends, Figures 1-7 and Table S1

Supplemental Figure 1. Validation of EYA4 stabilization and the mutually exclusive deletion patterns between TRIM69 and EYA4 in human cancer genomes. (A) *Top panel*: Western-blotting analyses examining abundance of endogenous EYA4 protein in Capan-2 (C-2) cells treated with Bortezomib (B, 200 nmol/L) or MG132 (M, 20 μmol/L) for 8 h. *Bottom panel*: Cellular ubiquitination assays comparing polyubiquitylation levels of EYA4 in Capan-2 (C-2) cells expressing GFP-tagged wild-type EYA4. PD, pull-down; Ni-NTA, Ni^{2+}-nitrilotriacetic acid. (B-D) Western-blotting analyses assessing levels of endogenous EYA4 protein in pancreatic patient-derived cancer cells (PDC, B), T3M-4 (T-4, C) cells as well as in human pancreatic ductal epithelial cells (HPDE, D) treated with Bortezomib (B, 200 nmol/L) or MG132 (M, 20 μmol/L) for 8 h. (E) Heatmap showing correlation between EYA4 and 40 E3 ligase-encoding genes in 12 pancreatic carcinoma cell lines from CCLE. *P* value was calculated by Pearson's rank correlation coefficient. (F-H) Heatmap showing correlation of EYA1 (F), EYA2 (G) and EYA3 (H) with 20 E3 ligase-encoding genes in 12 pancreatic carcinoma cell lines from CCLE as in (E). *P* value was calculated by Pearson's rank correlation coefficient. (I-M) Genetic alterations of EYA4-TRIM69 in liver (I), stomach (J), lung (K), kidney (L) and breast (M) cancer databases. The gene alteration percentages are shown.

Supplemental Figure 2. The role of TRIM69 and other memebers of TRIM family in EYA4 protein stability. (A and B) RT-qPCR (A) and western-blotting (B) analyses of EYA4 and P53 mRNA and protein expression in SW-1990 cells with or without TRIM69 shRNA (sh.TRIM69) transfection. Experiments were performed three times, each with quantitative RT-PCR in technical duplicate and real-time values were normalized to glyceraldehyde 3-phosphate dehydrogenase (GAPDH). Data are expressed as mean ± s.d.. $^{**}P < 0.01$. Two-sided Student's t test was used to calculate the *P* value. n.s, no significant. (C and D) RT-qPCR (C) and western-blotting (D) analyses of EYA4 and P53 mRNA and protein expression in SW-1990 cells with or without TRIM69 siRNA (si.TRIM69) transfection. Experiments were performed three times, each with quantitative RT-PCR in technical duplicate and real-time values were normalized to glyceraldehyde 3-phosphate dehydrogenase (GAPDH). Data are expressed as mean ± s.d. of three independent experiments. $^{**}P < 0.01$. Two-sided Student's t test was used to calculate the *P* value. n.s, no significant. (E) Western-blotting analyses comparing EYA4 protein levels in SW-1990 cells with or without TRIM13 siRNA (si.TRIM13), TRIM37 siRNA (si.TRIM37), TRIM25 siRNA

(si.TRIM25) and TRIM69 siRNA (si.TRIM69) transfection. (F-I) Western-blotting analyses examining the levels of TRIM13 (F), TRIM37 (G), TRIM25 (H) and TRIM69 (I) protein in SW-1990 cells with or without TRIM13 siRNA (si.TRIM13), TRIM37 siRNA (si.TRIM37), TRIM25 siRNA (si.TRIM25) and TRIM69 siRNA (si.TRIM69) transfection. (J) RT-qPCR analyses evaluating EYA4 mRNA levels in SW-1990 cells with or without TRIM13 siRNA (si.TRIM13), TRIM37 siRNA (si.TRIM37), TRIM25 siRNA (si.TRIM25) and TRIM69 siRNA (si.TRIM69) transfection. Experiments were performed three times, each with quantitative RT-PCR in technical duplicate and real-time values were normalized to glyceraldehyde 3-phosphate dehydrogenase (GAPDH). Data are expressed as mean ± s.d. **P*<0.05, ***P*<0.01 and ****P*<0.001. Two-sided ANOVA with Bonferroni post hoc t test correction was used to calculate the *P* value. (K) Western-blotting analyses examining EYA4 protein levels in SW-1990 cells with TRIM13 siRNA (si.TRIM13) transfection or TRIM13 siRNA (si.TRIM13) plus TRIM69 siRNA (si.TRIM69) cotransfection. (L) Western-blotting analyses detecting EYA4 protein abundance in GFP-EYA4-expressed SW-1990 cells with Flag-tagged wild-type TRIM69 transfection in the presence or absence of Bortezomib or MG132 treatment. V, Vehicle; D, DMSO; B, Bortezomib; M, MG132.

Supplemental Figure 3. TRIM69 degrades EYA4 and P53 independent of each other. (A) RT-qPCR analyses of *Eya4*, *Puma* and $p27^{Cdkn1b}$ gene expression in SW-1990 cells treated with or without RITA for 24 h. Experiments were performed three times, each with quantitative RT-PCR in technical duplicate and real-time values were normalized to glyceraldehyde 3-phosphate dehydrogenase (GAPDH). Data are expressed as mean ± s.d.. **P*<0.05, ***P*<0.01 and ****P*<0.001. Two-sided ANOVA with Bonferroni post hoc t test correction was used to calculate the *P* value. n.s, no significant. (B) Western-blotting analyses testing EYA4, PUMA and $p27^{CDKN1B}$ protein expression in SW-1990 cells treated with or without RITA for 24 h. (C) Western-blotting analyses testing the levels of endogenous EYA4 and P53 protein in sg.TRIM69-transduced SW-1990 cells with scrambled shRNA (Scr) or P53 shRNA (sh.P53) transfection. (D) Western-blotting analyses examining P53 protein expression in SW-1990 (S-1) and T3M-4 (T-4) cells with or without sg.TRIM69 transduction. (E) Cycloheximide (CHX) pulse-chase experiments determining turnover of endogenous EYA4 protein in SW-1990 cells with or without sg.TRIM69 transduction in the presence or absence of 20 μg/mL CHX treatment for the indicated times, respectively. (F) Cellular ubiquitination assays comparing polyubiquitylation levels of EYA4 in sg.TRIM69-transduced SW-1990 cells with full-length (FL) or

RING finger domain truncation (ΔR) TRIM69 reconstitution. (G) Cellular ubiquitination assays comparing the K48- or K63-linked polyubiquitylation levels of EYA4 in GFP-EYA4-expressed SW-1990 cells with or without Flag-tagged wild-type TRIM69 introduction. (H) Coimmunoprecipitation assay assessing the interaction between EYA4 and TRIM69 in SW-1990 cells coexpressed GFP-EYA4 and Flag-tagged wild-type TRIM69.

Supplemental Figure 4. TRIM69 is upregulated in human PDAC. (A) Pan-cancer analyses of human *Trim69*, *Ctnnb1l* and *Id2* expression in different tumor types from The Cancer Genome Atlas database. (B) Relative expression of *Trim69*, *Ctnnb1l* and *Id2* expression in normal pancreatic tissues (n = 171) and pancreatic cancer tissues (n = 179) from The Cancer Genome Atlas. (C) Comparison of *Trim69* expression between normal pancreatic tissues and pancreatic cancer samples from three independent cohorts in the Oncomine database. (D) Correlation between TRIM69 expression and clinicopathological features of PDAC patients from Cohort 2.

Supplemental Figure 5. TRIM69 impairs the EYA4-driven tumor suppression. (A) *Left panel*: Western-blotting analyses detecting α-PKA protein expression in SW-1990 cells with or without PKA siRNA (si. PKA) transfection. *Right panel*: Western-blotting analyses evaluating phosphorylation of β-catenin at Ser675 in SW-1990 cells with GFP-EYA4 or PKA siRNA (si. PKA) transfection or GFP-EYA4 plus Flag-tagged wild-type TRIM69 cotransfection. (B) Representative immunfluorescence images of nuclear β-catenin inclusion in SW-1990 cells with GFP-EYA4 transfection or GFP-EYA4 plus Flag-tagged wild-type TRIM69 cotransfection in the presence or absence of Wnt-3a (20 ng/mL) stimulation. Scale bar = 25 μm. (C) Representative images of subcutaneous xenografts formed by SW-1990 and Capan-2 cells with GFP-EYA4 transfection or GFP-EYA4 plus Flag-tagged wild-type TRIM69 cotransfection, respectively. (D) Tumor weight of subcutaneous xenografts excised from the mice bearing SW-1990 or Capan-2 tumors at day 35. Data are presented as mean ± s.d. $^{**}P < 0.01$. Two-sided ANOVA with Bonferroni post hoc t test correction was used to calculate the P value. (E) Representative immunfluorescence images and quantification of Ki-67 staining in subcutaneous xenografts formed by SW-1990 and Capan-2 cells with GFP-EYA4 transfection or GFP-EYA4 plus Flag-tagged wild-type TRIM69 cotransfection, respectively. Data are presented as mean ± s.d. of at least six independent experiments. Scale bar = 100 μm.

Supplemental Figure 6. Depletion of TRIM69 induces death and suppresses

tumor growth in EYA4-deficient PDAC cells. (A) MTT assays assessing cell viability of the indicated SW-1990 (S-1), Capan-2 (C-2) and T3M-4 (T-4) cells with TRIM69 siRNA (si.TRIM69) transfection. Data are presented as mean ± s.d. $^{*}P < 0.05$ and $^{**}P < 0.01$. Two-sided ANOVA with Bonferroni post hoc t test correction was used to calculate the *P* value. Un, untrasfected. (B) Western-blotting analyses examining the abundance of TRIM69 protein expression in SW-1990 (S-1) or Capan-2 (C-2) cells and T3M-4 (T-4) cells with control siRNA (si.Ctrl) or TRIM69 siRNA (si.TRIM69) transfection, and determining levels of EYA4 protein in EYA4 shRNA (sh.EYA4)-expressed T-4 cells (T-4/sh.EYA4). (C) MTT assays measuring cell viability of the indicated SW-1990 (S-1), Capan-2 (C-2) and T3M-4 (T-4) cells with TRIM69 shRNA (sh.TRIM69) transfection. Data are presented as mean ± s.d. $^{**}P < 0.01$ and $^{***}P < 0.001$. Two-sided ANOVA with Bonferroni post hoc t test correction was used to calculate the *P* value. (D) Western-blotting analyses examining the abundance of TRIM69 protein expression in SW-1990 (S-1) or Capan-2 (C-2) cells and T3M-4 (T-4) cells with scrambled shRNA (Scr) or TRIM69 (sh.TRIM69) transfection, and assessing levels of EYA4 protein in EYA4 shRNA (sh.EYA4)-expressed T-4 cells (T-4/sh.EYA4). (E-H) Tumor weight and representative images of subcutaneous xenografts formed by SW-1990 (S-1, E) or Capan-2 (C-2, F), T3M-4 (T-4/−, G) as well as EYA4 shRNA (sh.EYA4)-expressed T3M-4 (T-4/sh.EYA4, H) cells with or without sg.TRIM69 transduction from nude mice. Data are presented as mean ± s.d. $^{*}P < 0.05$. Two-sided Student's t test was used to calculate the *P* value.

Supplemental Figure 7. ERK2 directly interacts with and phosphorylates EYA4, which is essential for polyubiquitination and turnover of EYA4 by TRIM69 as well as activation of β-catenin/ID2 pathway. (A) Coimmunoprecipitation assay testing the interaction between EYA4 and endogenous ERK1/2 in GFP-EYA4-expressed SW-1990 cells. (B) Recognition of D-site motif in EYA4 protein and other known ERK2 substrates for comparison. (C) Western-blotting analyses comparing levels of p-ERK1/2 protein expression in SW-1990 cells with 100 ng/mL EGF (E) stimulation. D, DMSO. (D) Cellular ubiquitination assays examining polyubiquitylation levels of EYA4 in GFP-EYA4-expressed SW-1990 cells cotransfected with vectors bearing the Myc-tagged $MEK1^{CA}$ and ERK2 proteins. (E) Western-blotting analyses detecting abundance of p-ERK1/2 protein in SW-1990 cells with 10 μM SCH772984 (SCH) administration. D, DMSO. (F) Western-blotting analyses determining levels of ERK2 protein in SW-1990 cells transfected with control siRNA (si.Ctrl) or ERK2 siRNA (si.ERK2). (G) Cellular ubiquitination assays

assessing polyubiquitylation levels of EYA4 in GFP-EYA4-expressed SW-1990 cells with or without Flag-tagged wild-type TRIM69 introduction in the presence of ERK2 siRNA (si.ERK2) transfection. (H) Coimmunoprecipitation assay evaluating Ser phosphorylation of EYA4 in GFP-EYA4-expressed SW-1990 cells with or without EGF (100 ng/mL) stimulation in the presence or absence of SCH772984 (SCH) administration. (I) Coimmunoprecipitation assay determining Ser phosphorylation of EYA4 in GFP-EYA4-expressed SW-1990 cells with or without 100 ng/mL EGF (E) stimulation in the presence or absence of ERK2 siRNA (si.ERK2) transfection. (J) Representative immunfluorescence images of nuclear β-catenin inclusion in Wnt3a-stimulated SW-1990 cells with empty vector (EV), GFP-tagged wild-type EYA4 (WT) or mutant EYA4 Ser37E (S37E) transfection. Scale bar = 25 μm. (K) RT-qPCR and western-blotting analyses measuring the levels of *Id2* gene and protein expression in Wnt3a-stimulated SW-1990 cells with empty vector (EV), GFP-tagged wild-type EYA4 (WT) or mutant EYA4 Ser37E (S37E) transfection. Data are expressed as mean ± s.d. of three independent experiments. *$P < 0.05$. Two-sided ANOVA with Bonferroni post hoc t test correction was used to calculate the *P* value.

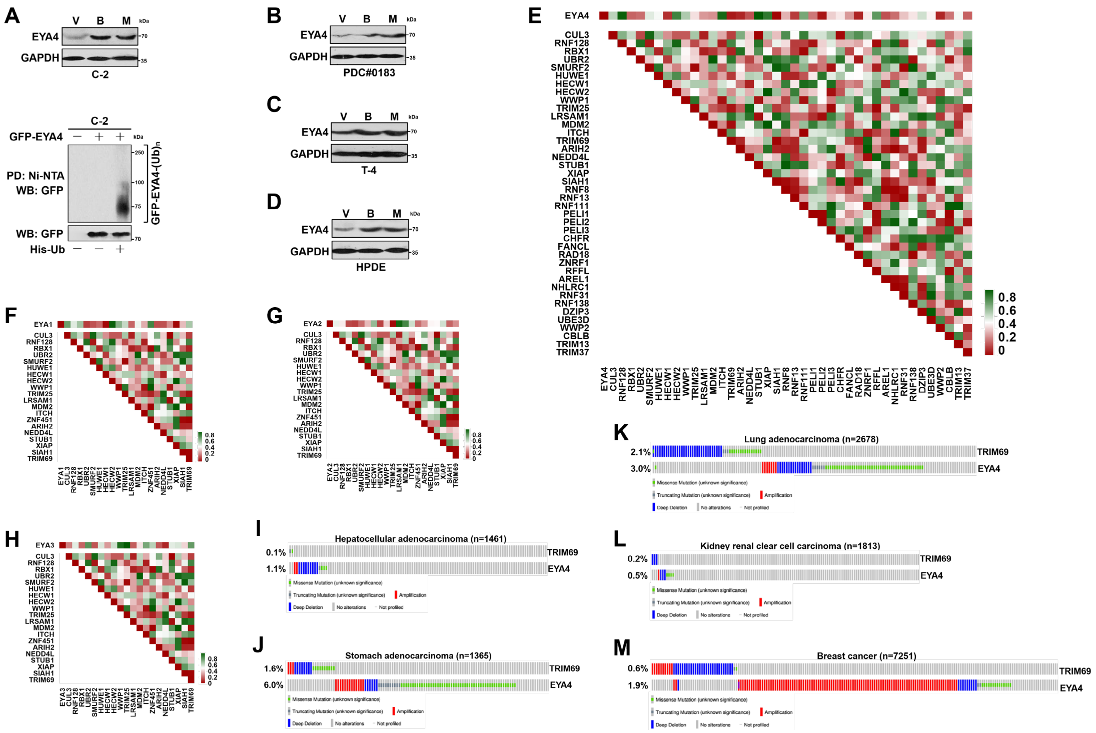

A
B
C
D
E
F
G
H
I
J
K
L
M
EYA4
GAPDH
C-2
PDC#0183
T-4
HPDE
V B M
GFP-EYA4
PD: Ni-NTA
WB: GFP
His-Ub
GFP-EYA4-(Ub)n
Lung adenocarcinoma (n=2678)
Hepatocellular adenocarcinoma (n=1461)
Kidney renal clear cell carcinoma (n=1813)
Stomach adenocarcinoma (n=1365)
Breast cancer (n=7251)
TRIM69

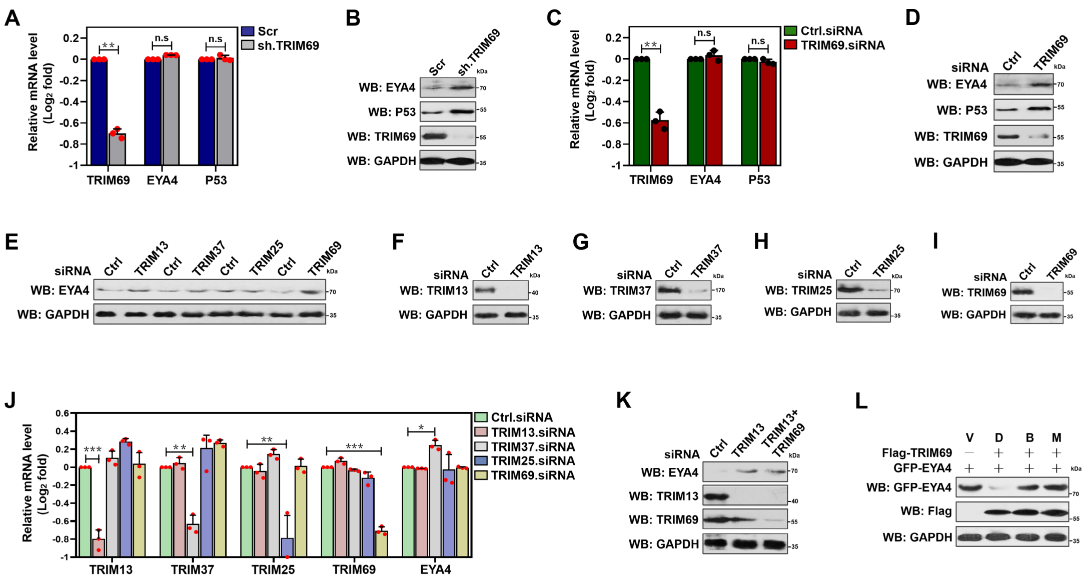
A
Relative mRNA level (Log2 fold)
Scr
sh.TRIM69
TRIM69
EYA4
P53
B
Scr
sh.TRIM69
WB: EYA4
WB: P53
WB: TRIM69
WB: GAPDH
C
Ctrl.siRNA
TRIM69.siRNA
D
siRNA
Ctrl
TRIM69
E
TRIM13
TRIM37
TRIM25
F
G
H
I
J
TRIM13.siRNA
TRIM37.siRNA
TRIM25.siRNA
K
TRIM13+TRIM69
WB: TRIM13
L
V
D
B
M
Flag-TRIM69
GFP-EYA4
WB: GFP-EYA4
WB: Flag
kDa

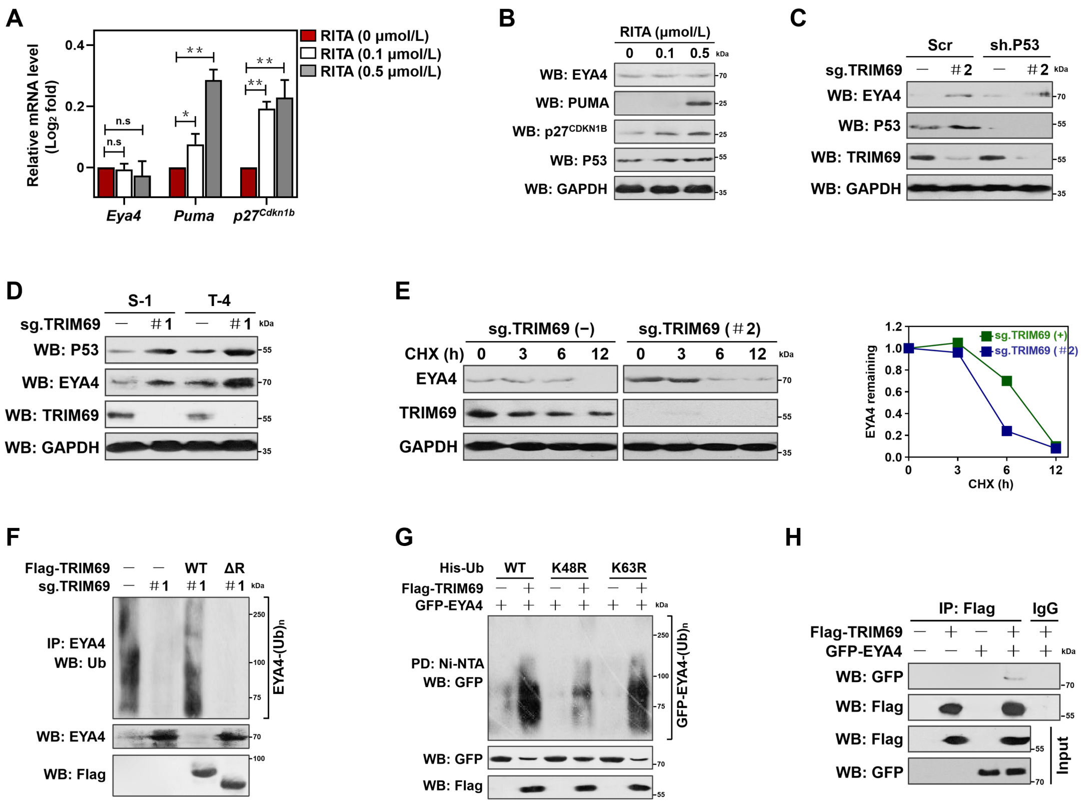

A
B
C
D
E
F
G
H
RITA (0 µmol/L)
RITA (0.1 µmol/L)
RITA (0.5 µmol/L)
Relative mRNA level (Log2 fold)
Eya4
Puma
p27Cdkn1b
RITA (µmol/L)
WB: EYA4
WB: PUMA
WB: p27CDKN1B
WB: P53
WB: GAPDH
Scr
sh.P53
sg.TRIM69
WB: TRIM69
S-1
T-4
sg.TRIM69 (−)
sg.TRIM69 (＃2)
CHX (h)
EYA4
TRIM69
GAPDH
EYA4 remaining
sg.TRIM69 (+)
sg.TRIM69 (＃2)
Flag-TRIM69
IP: EYA4
WB: Ub
EYA4-(Ub)n
WB: Flag
His-Ub
WT
K48R
K63R
GFP-EYA4
PD: Ni-NTA
WB: GFP
GFP-EYA4-(Ub)n
IP: Flag
IgG
Input
kDa

A

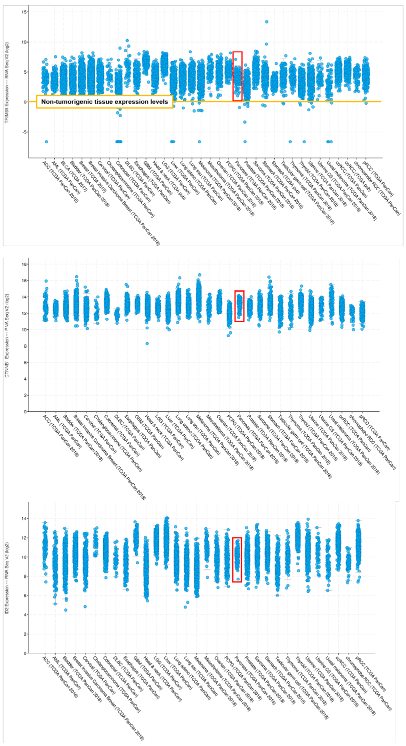

B

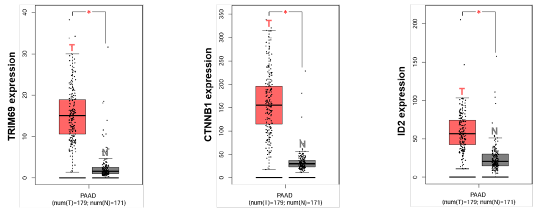

C

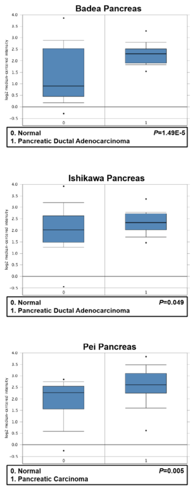

D

Variable		TRIM69		*P*
		Low	High	Value
Age	≥ 50	30	63	0.445
	< 50	9	13	
Gender	Male	17	54	0.004
	Female	22	22	
Diameter (cm)	≥ 0.4	4	4	0.323
	< 0.4	35	72	
Location	Head	36	69	0.787
	Other	3	7	
Differentiation	Poor	21	48	0.339
	Well	18	28	
SMV invasion	Yes	35	47	0.002
	No	4	29	
TNM stage	I/II	31	56	0.822
	III/IV	8	20	
Chemotherapy	Yes	32	69	0.178
	No	7	7	
Survival	Death	8	57	0.005
	Survival	31	19	

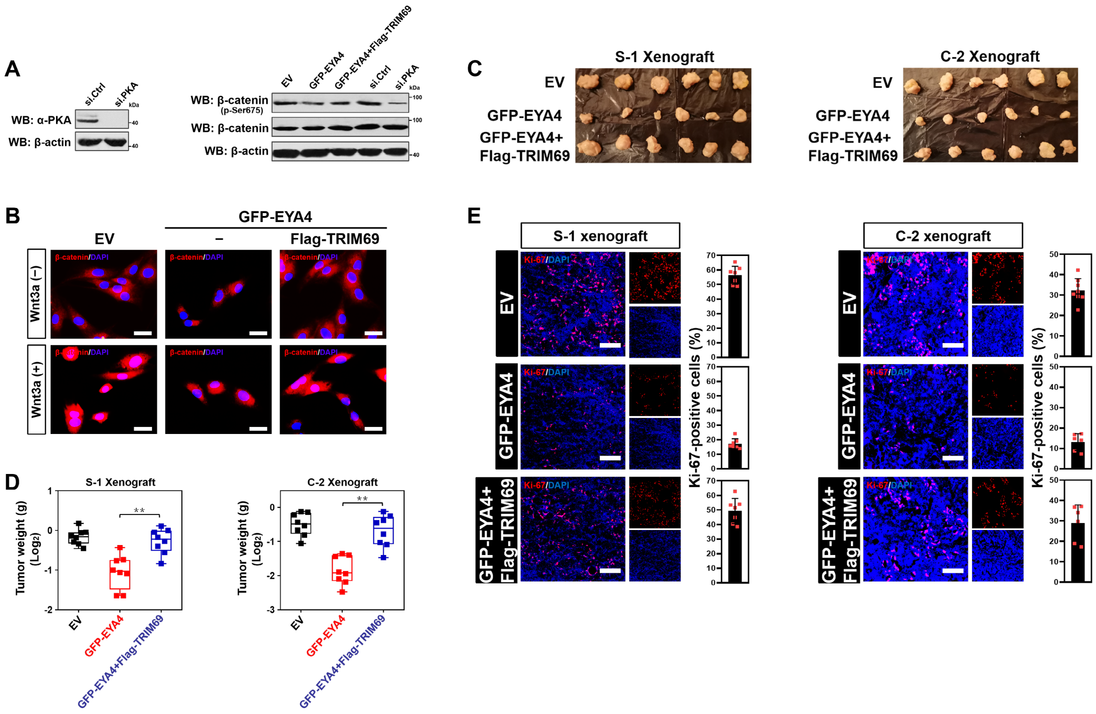

A
si.Ctrl
si.PKA
WB: α-PKA
WB: β-actin
EV
GFP-EYA4
GFP-EYA4+Flag-TRIM69
si.Ctrl
si.PKA
WB: β-catenin (p-Ser675)
WB: β-catenin
WB: β-actin
B
EV
GFP-EYA4
–
Flag-TRIM69
Wnt3a (–)
Wnt3a (+)
β-catenin/DAPI
C
S-1 Xenograft
C-2 Xenograft
EV
GFP-EYA4
GFP-EYA4+ Flag-TRIM69
D
S-1 Xenograft
C-2 Xenograft
Tumor weight (g) (Log2)
EV
GFP-EYA4
GFP-EYA4+Flag-TRIM69
E
S-1 xenograft
C-2 xenograft
Ki-67/DAPI
Ki-67-positive cells (%)

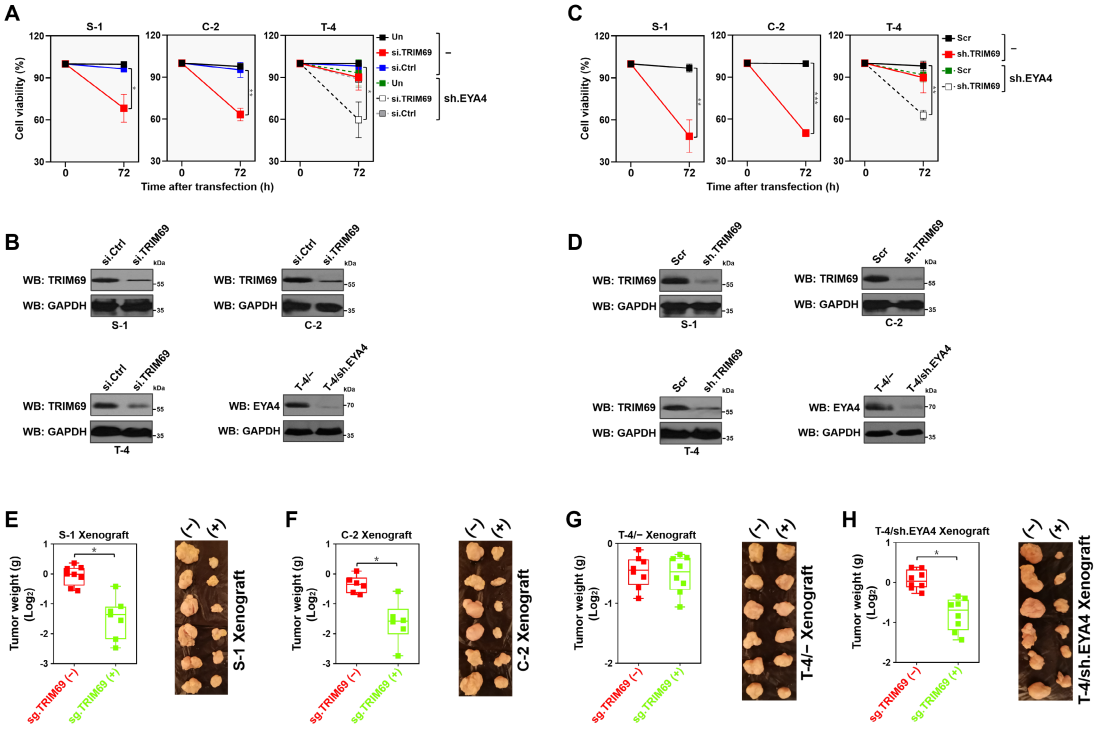
A
S-1
C-2
T-4
Cell viability (%)
Time after transfection (h)
Un
si.TRIM69
si.Ctrl
sh.EYA4
B
si.Ctrl
si.TRIM69
WB: TRIM69
WB: GAPDH
WB: EYA4
T-4/−
T-4/sh.EYA4
kDa
C
Scr
sh.TRIM69
D
E
S-1 Xenograft
F
C-2 Xenograft
G
T-4/− Xenograft
H
T-4/sh.EYA4 Xenograft
Tumor weight (g) (Log2)
sg.TRIM69 (−)
sg.TRIM69 (+)

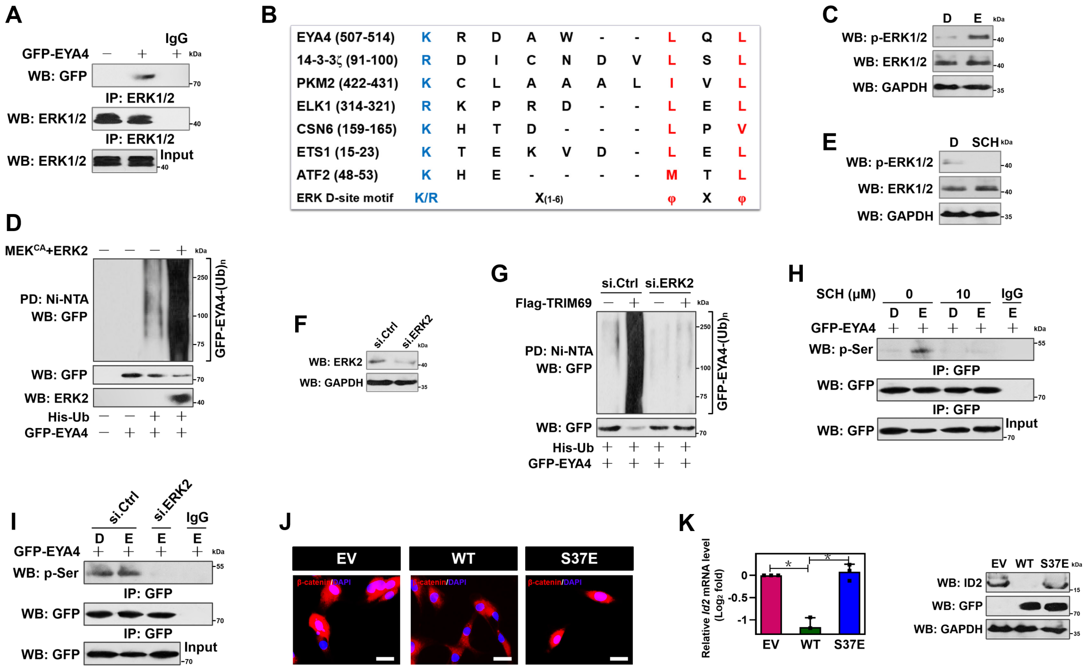

A
GFP-EYA4
IgG
WB: GFP
IP: ERK1/2
WB: ERK1/2
IP: ERK1/2
WB: ERK1/2
Input
B
EYA4 (507-514) K R D A W - - L Q L
14-3-3ζ (91-100) R D I C N D V L S L
PKM2 (422-431) K C L A A A L I V L
ELK1 (314-321) R K P R D - - L E L
CSN6 (159-165) K H T D - - - L P V
ETS1 (15-23) K T E K V D - L E L
ATF2 (48-53) K H E - - - - M T L
ERK D-site motif K/R X(1-6) φ X φ
C
D E
WB: p-ERK1/2
WB: ERK1/2
WB: GAPDH
D
MEKCA+ERK2
PD: Ni-NTA
WB: GFP
GFP-EYA4-(Ub)n
WB: GFP
WB: ERK2
His-Ub
GFP-EYA4
E
D SCH
WB: p-ERK1/2
WB: ERK1/2
WB: GAPDH
F
si.Ctrl
si.ERK2
WB: ERK2
WB: GAPDH
G
si.Ctrl si.ERK2
Flag-TRIM69
PD: Ni-NTA
WB: GFP
GFP-EYA4-(Ub)n
WB: GFP
His-Ub
GFP-EYA4
H
SCH (μM) 0 10 IgG
D E D E E
GFP-EYA4
WB: p-Ser
IP: GFP
WB: GFP
IP: GFP
WB: GFP
Input
I
si.Ctrl
si.ERK2
IgG
D E E E
GFP-EYA4
WB: p-Ser
IP: GFP
WB: GFP
IP: GFP
WB: GFP
Input
J
EV
WT
S37E
K
Relative Id2 mRNA level (Log2 fold)
EV WT S37E
EV WT S37E
WB: ID2
WB: GFP
WB: GAPDH

Supplemental Table 1 Clinicopathological features in PDAC patients (n=115)

Variable	No. of patients	%
Gender		
Male	73	63.5
Female	42	36.5
Age (years)		
<60	51	44.3
≥60	64	55.7
Tumor size		
≤40mm	87	75.6
>40mm	28	24.4
Tumor location		
Head	90	78.3
Body	17	14.8
Tail	8	6.9
Lymphatic metastasis		
Negative	69	60.0
Positive	46	40.0
TNM stage		
Ⅰ	32	27.8
Ⅱ	59	51.3
Ⅲ	15	13.0
Ⅳ	9	7.9
Chemotherapy		
Yes	94	81.7
No	21	18.3
TRIM69 expression		
High	76	66.1
Low	39	33.9
